# Emerging MoS_2_ Wafer-Scale Technique for Integrated Circuits

**DOI:** 10.1007/s40820-022-01010-4

**Published:** 2023-01-18

**Authors:** Zimeng Ye, Chao Tan, Xiaolei Huang, Yi Ouyang, Lei Yang, Zegao Wang, Mingdong Dong

**Affiliations:** 1 College of Materials Science and Engineering, Sichuan University, Chengdu, 610065 People’s Republic of China; 2 State Key Laboratory of Solidification Processing, Center of Advanced Lubrication and Seal Materials, Northwestern Polytechnical University, Xi’an, 710072 People’s Republic of China; 3 Interdisciplinary Nanoscience Center, Aarhus University, 8000 Aarhus C, Denmark

**Keywords:** Wafer-scale growth, Molybdenum disulfide, Gas deposition, Integrated circuits

## Abstract

As an outstanding representative of layered materials, molybdenum disulfide (MoS_2_) has excellent physical properties, such as high carrier mobility, stability, and abundance on earth. Moreover, its reasonable band gap and microelectronic compatible fabrication characteristics makes it the most promising candidate in future advanced integrated circuits such as logical electronics, flexible electronics, and focal-plane photodetector. However, to realize the all-aspects application of MoS_2_, the research on obtaining high-quality and large-area films need to be continuously explored to promote its industrialization. Although the MoS_2_ grain size has already improved from several micrometers to sub-millimeters, the high-quality growth of wafer-scale MoS_2_ is still of great challenge. Herein, this review mainly focuses on the evolution of MoS_2_ by including chemical vapor deposition, metal–organic chemical vapor deposition, physical vapor deposition, and thermal conversion technology methods. The state-of-the-art research on the growth and optimization mechanism, including nucleation, orientation, grain, and defect engineering, is systematically summarized. Then, this review summarizes the wafer-scale application of MoS_2_ in a transistor, inverter, electronics, and photodetectors. Finally, the current challenges and future perspectives are outlined for the wafer-scale growth and application of MoS_2_.

## Highlights

- This review summarized the state of the art of MoS_2_ from their controllable growth and potential application in integrated circuit.
- The influence of promoter, substrate, pressure, catalyst and precursor on the nucleation and growth are discussed.
- The current challenges and future perspectives of wafer-scale MoS_2_ are outlined from the materials and device applications.

## Graphical abstract

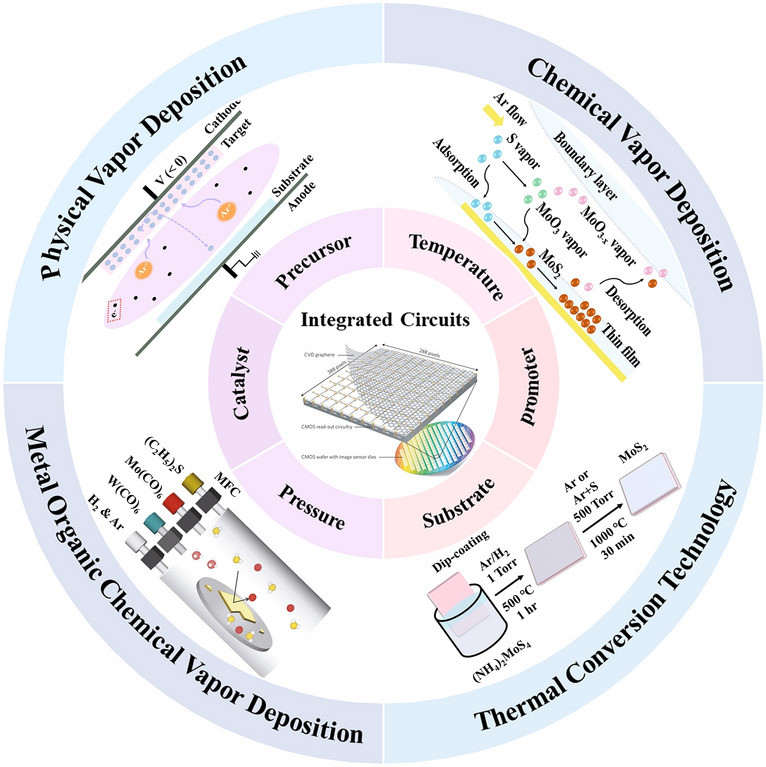

## Introduction

In the "post-Moore's Law" era, new materials with smaller volumes and better performance are expected to innovate existing electronic and optoelectronic technologies to meet the higher performance requirements of human progress in electronic devices [1–5]. Two-dimensional (2D) nanostructured materials have a high surface-to-volume ratio compared to their bulk materials, which offers a strong surface state in electrical transmission, leading to more negligible scattering and higher carrier mobility [6]. Apart from this, their atomic-level thickness, excellent performance, and mature device fabrication technology make them become ideal semiconductor materials as well [7, 8]. However, most articles about preparing large-scale, high-quality films at present need to meet the requirements of integrated, flexible electronic equipment, which define the films as single crystals with large sizes [9, 10]. Therefore, the acquisition of low defect-density and continuous film has gradually become a research focus in 2D materials.

Among various 2D materials, molybdenum disulfide (MoS_2_) is one of the representative materials whose outstanding mechanical, optical, and electronic properties endow it with great potential for future applications in noble nanoscale electronic and optoelectronic devices [3, 11–13]. Based on previous reports, the physical properties of intrinsic MoS_2_ are closely related to the film thickness [14, 15]. The single-layer MoS_2_ film is a semiconductor with a direct band gap of 1.83 eV, which can produce strong photoluminescence and electroluminescence [16, 17]. At present, the threshold voltage of single-layer MoS_2_ synthesized by chemical vapor deposition technology is less than − 50 V, showing prominent n-type conductivity characteristics [16, 18]. This heavy doping effect can be mainly attributed to the large number of charge traps generated by the amorphous SiO_2_/Si substrate. In addition, the theoretical carrier mobility of the single-layer MoS_2_ can reach ~ 410 cm^2^ V^−1^ s^−1^, and the on–off ratio is 10^8^ [19]. However, experimentally, the mobility can only reach 90 cm^2^ V^−1^ s^−1^ [20]. Even at low temperatures, its mobility can hardly be increased to 200 cm^2^ V^−1^ s^−1^, far poorer than the theoretically predicted value [21]. Besides, contact engineering, such as van der Waals contact, has shown potential improved the electrical property of MoS_2_ [2, 6, 22–24]. Even though the present electrical property of MoS_2_ is still much lower than its predicted value. It is predicted that the short-range scattering caused by the defects of the film structure severely limits the performance of the CVD-MoS_2_ film. It is necessary to explore and optimize the preparation technology of the MoS_2_ sheet from the study of material growth mechanism and process research [25]. To realize its practical application, finding ways for controllable preparation of high-quality wafer-scale single crystal MoS_2_ film is a prerequisite. The maximum size of single crystal MoS_2_ has not yet reached the centimeter level, so it is still a challenge to manufacture large-scale MoS_2_-based atomic-thin integrated circuits with high device density and performance. But research on the synthesis of MoS_2_ is ongoing, and new progress, including growth mechanism and impact factors, keeps emerging.

**Fig. 1:**
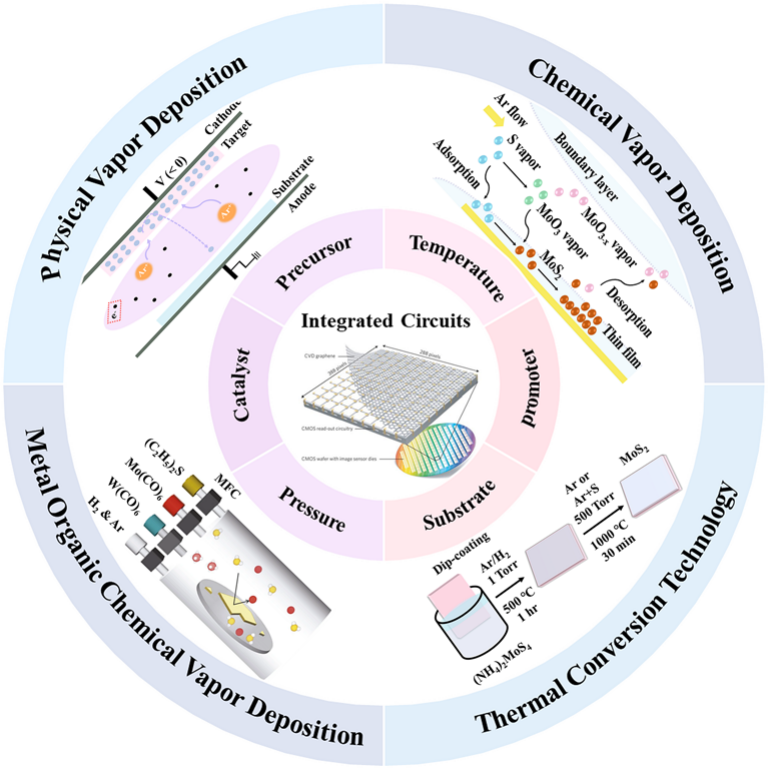
Scope of this review

This article will start with specific experimental methods, including physical vapor deposition, chemical vapor deposition, magnetron sputtering, etc., and then mainly focus on factors such as precursor type, growth pressure, carrier gas, and catalyst effect to summarize the structure and properties of MoS_2_ film prepared based on CVD-systems, finally perspectives on their future development trend. The scope of this review is shown in Fig. 1.

## Growth of the Wafer-Scale MoS_2_ Film

### Physical Vapor Deposition Technology

**Fig. 2:**
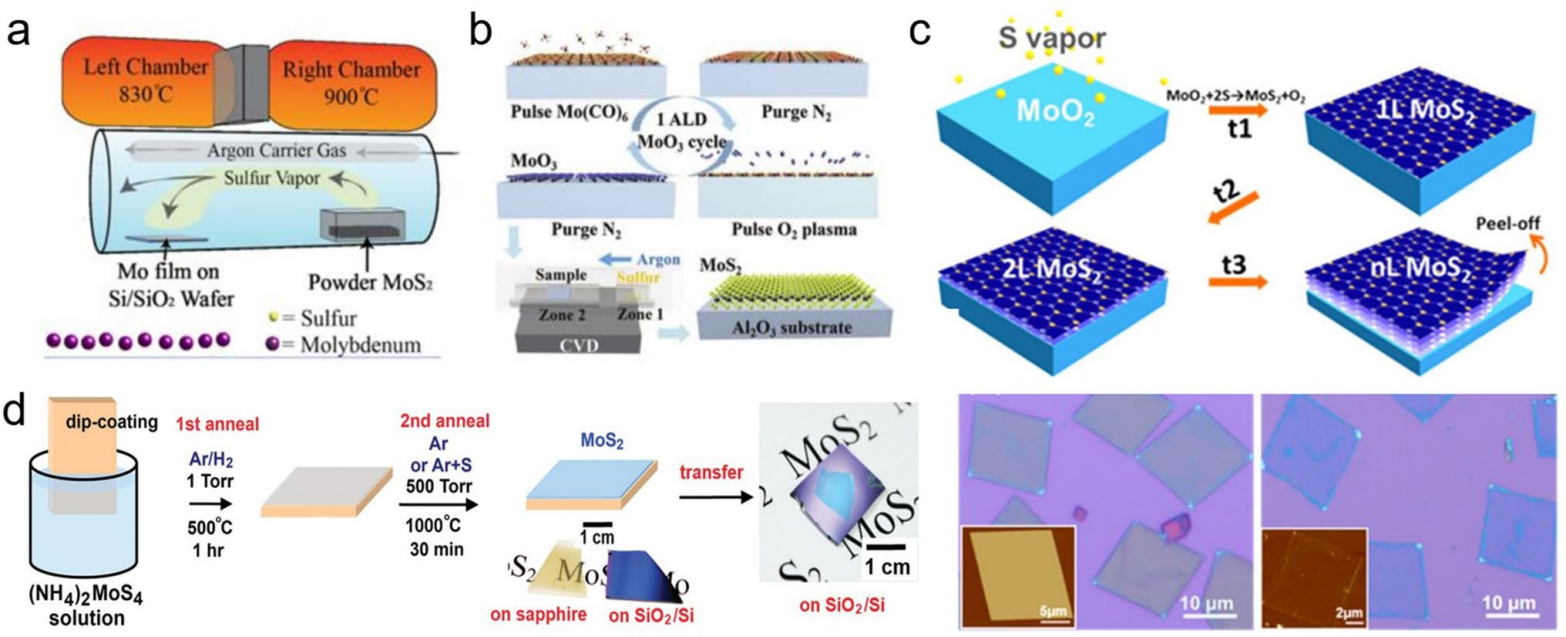
The film morphology and corresponding characteristics obtained by thermal conversion method. **a** Growth the MoS_2_ film by directly thermal conversion of metal Mo film [29], Copyright 2019, Institute of Physics. **b** Growth of the MoS_2_ film by directly thermal conversion of MoO_3_ film [28], Copyright 2017, WILEY–VCH. **c** Growth of the MoS_2_ film by directly thermal conversion of MoO_2_ film [30], Copyright 2013, American Chemical Society. **d** Growth of the MoS_2_ film by directly thermal conversion of (NH_4_)_2_MoS_4_ [31], Copyright 2012, American Chemical Society

Physical Vapor Deposition (PVD) technology, such as magnetron sputtering and thermal evaporation, can easily obtain ultra-thin and large-area transition-metal dichalcogenides (TMDCs) materials on different substrates and does not require high temperatures [26]. This can be attributed to the fact that PVD inputs a large amount of energy to the surface of the film so that the incident atoms still have a high surface migration rate at low-temperature conditions, then the large-scale film can be easily acquired. In addition, PVD can maintain interface cleanliness and precisely control the film thickness in an ultra-high vacuum environment. By magnetron sputtering, Muratore et al. reported the growth of MoS_2_ film with large-area and low-defect density where its photoluminescence intensity is comparable with mechanically exfoliated MoS_2_ [27]. The researcher found that some factors need to be optimized, for example, the power of the sputtering source, the intensity of the magnetic field, and the electrostatic field of the adjacent substrate to change the sputtering atomic beam energy. The sputtering atomic beam energy should be approximately 8 eV, lower than the lowest energy threshold of defect formation. In addition, researchers also pointed out that by choosing amorphous SiO_2_ or (002) oriented graphite as the growth substrate, the film surface, and interface pollution can be effectively improved. However, this method has inherent defects within film uniformity, deposition repeatability, and process stability. More mature processes and broader applications need to be further explored. To minimize the above problem, the method of physical vapor deposition-assisted CVD is reported, as shown in Fig. 2. For example, electron beam evaporation has been proven effective for the large-scale and controllable growth of TMDCs on various substrates [28]. It is proved that the thickness of the MoS_2_ sheet can be determined by the thickness of the Mo film or MoO_x_ film, which is then converted to control the evaporation rate and time of the PVD process.

### Thermal Conversion Technology

The thermal conversion method refers to the preparation of 2D materials by direct vulcanization reaction of the film pre-deposited on a substrate, as shown in Fig. 2. Compared with the chemical vapor deposition technology, the quality of the 2D-TMDCs film prepared by the sulfuration reaction is mainly controlled by the quality of the pre-deposited film so that the film generally has a higher in-plane uniformity. In addition to the preparation of a single-component thin film, the sulfuration reaction can also be used for the direct synthesis of 2D-heterojunction. Jung et al. pre-prepared patterned metal W and metal Mo films by photolithography and then applied them to a sulfur-rich environment managed to synthesize cm-level 2D MoS_2_/WS_2_ heterojunction [32]. When applying it to a diode optoelectronic device, the measured current on–off ratio exceeds 1000 (at ± 0.5 V), showing prominent interface rectification characteristics. Typically, the pre-deposited film is the metal element-based film, for example, pure Mo film, oxide Mo film, or others. Thus, the following sulfuration has a little bit different, which will be discussed later.

#### Directly Sulfuration of Metal Mo Film

Directly sulfuration of metal Mo film is a film growth technology that produces wafer-scale 2D materials with high carrier mobility and high substrate coverage. It can achieve doping and patterning processes as well as heterostructure fabricating. Zhan et al*.* reported the growth of MoS_2_ film by directly sulfurating the metal Mo film by using sulfur powder as the S-source [33]. Due to a large amount of cationic vacancy, it was found the MoS_2_ film showed p-type electrical properties with low field modulation. Robertson et al*.* used MoS_2_ powder as S-source, which was annealed by 900 °C and released stable sulfur vapor. By controlling the thickness of the metal Mo film, it was found that the thickness of the MoS_2_ can be controllable in the range from 1-layer to 4-layer. The size of the MoS_2_ film can be as large as 2.5 cm, as shown in Fig. 2a [29]. Furthermore, the researchers found that in the range of 1–5 layers, the relationship between the pre-deposited Mo film and the resulting MoS_2_ film is about 1:2. By studying the electrical property of the MoS_2_-based transistor, it was found that the carrier mobility is about 0.05 cm^2^ V^−1^ s^−1^, which still need further optimization. Besides the growth of the pure MoS_2_, direct sulfuration has also usually been applied to grow the doped MoS_2_ film due to its easily deposited the doped metal film, such as the Nb-doped MoS_2_ [34, 35].

#### Direct Sulfurization of Molybdenum Oxide

Using MoO_2_, MoS_2_ can be epitaxially grown into an ultra-thin continuous film at high temperatures. The pre-deposited MoO_2_ film can be converted to a quasi-single crystal MoS_2_ film after sulfuration. However, the direct sulfuration of MoO_2_ film is prone to generate small-angle grain boundaries and texture, where both of the phenomena get more apparent with the increase of the precursor film thickness. The tendency to produce textured MoS_2_ films may be caused by high-density defects introduced during the epitaxial process of MoO_2_ film. To eliminate possible defects, Xu et al*.* proposed a capping layer annealing process (CLAP), which can minimize defects. And further experimental results show that the MoS_2_ film treated with CLAP is no longer a textured film, and its carrier mobility can be increased to 6.3 cm^2^ V^−1^ s^−1^, which is about 15 times that of the previous textured film [36, 37]. Also, the adhesion between MoO_2_ and MoS_2_ is weak. Wang et al. prepared MoS_2_ films with crystallized MoO_2_ microplates as the Mo-containing precursor. Based on the weak adhesion, they achieved an average domain size of about 10 μm MoS_2_ film with high quality (carrier mobility is 0.1–10 cm^2^ V^−1^ s^−1^) [18].

MoO_3_ has a relatively low melting and evaporation temperature (about 700 °C). Lee et al*.* proved that MoO_3_ is a suitable Mo-containing precursor for MoS_2_ growth using CVD. Similar to MoO_2_ film, the number of layers of the MoS_2_ film obtained by MoO_3_ is controlled by the pre-deposited precursor film [15]. Shi et al*.* achieved wafer-scale high-quality MoS_2_ film with high uniformity by sulfurizing the MoO_3_ film, which is pre-deposited by ALD using Mo(CO)_6_ as the Mo-source, as shown in Fig. 2b [28]. The advance of this method is that the cycling number could easily control the thickness of the pre-deposited MoO_3_ during the growth. By continuous optimization of growth conditions, the continuous film size was expanded to 2 inches, and the number of layers was strictly controllable from 1 to 4 layers. It was found that the MoS_2_ film grown by this method would have the carrier mobility of 0.76 and 5.9 cm^2^ V^−1^ s^−1^ for monolayer and four-layer, respectively. Similarly, this method has also been applied to grow other TMDCs films. Kim et al. obtained high-quality WS_2_ film with carrier mobility of 3.9 cm^2^ V^−1^ s^−1^ based on the same method [32]. The advantage of MoO_3_ film is that it is first converted to α-MoO_3_ during sulfuration, which contributes to forming a favorable MoS_2_-sapphire interface, thereby reducing Coulomb Scattering.

#### Conversion of Mo-Containing Salt

Solution spin coating is a method that configures a homogeneous solution with Mo-containing precursor (soluble salt), then spin-coats it on the substrate surface, and finally sulfur the precursor film [38]. The method can quickly obtain a continuous uniform MoS_2_ film and reduce the number of film layers by diluting the precursor solution and accelerating the rotating speed. However, it also tends to cause high-density nucleation to form polycrystalline. An improved method uses the spin-coated substrate as the precursor and places it on the opposite side of the growth substrate, which not only avoids high-density nucleation but also guarantees a sufficient reaction atoms supply, supporting the growth of the wafer-scale 2D film. Ammonium thiomolybdate ((NH_4_)_2_MoS_4_) can be used as both Mo-containing and S-containing precursors. In 2012, it was reported that large-scale MoS_2_ film could be grown in a single-temperature-zone tube furnace, significantly simplifying the experimental system [33]. During the growth, there was a two-step pyrolysis process. In the first step, ammonium thiomolybdate ((NH_4_)_2_MoS_4_) was pyrolyzed and converted into MoS_3_ in an N_2_ atmosphere at around 120–360 °C. In the second step, the temperature was increased (> 800 °C), realizing the conversion from MoS_3_ to MoS_2_. It is worth noting that the conversion process from (NH_4_)_2_MoS_4_ to MoS_2_ involves a multi-step reaction and is susceptible to O_2_. Therefore, H_2_ needs to be introduced to avoid the oxidation of MoS_2_. Further studies have shown that the introduction of H_2_ can directly achieve a one-step conversion, and the conversion temperature can be reduced to about 425 °C, which significantly saves energy. In terms of the facile method, Lim et al. demonstrated the roll-to-roll growth of the MoS_2_ film by using the (NH_4_)_2_MoS_4_ as the precursor and exhibited its potential application in transistors, photodetectors, and electrocatalysis [39]. It was reported that the carrier mobility is in the range of 0.022 ~ 0.6 cm^2^ V^−1^ s^−1^. Thus further improvement is still needed [39, 40]. Due to the solubility of the salt, a similar method has also been applied to grow WS_2_ film, doped MoS_2_, and even the alloy MoWS_2_ film [41]. However, the drawback of this method is that the formed film usually has a small grain size whose strong carrier scattering results in low electrical properties. Recently, it has been demonstrated that the grain size could be improved by tuning the property of the substrate and the precursor morphology [42, 43].

#### Inhibitor-Utilizing Atomic Layer Deposition (iALD) of Mo(CO)_6_

Mo(CO)_6_ is a coordination compound that can be used as a Mo-containing precursor to support the growth of MoS_2_ film. Jeon et al. [44] obtained high quality MoS_2_ film with outstanding carrier mobility (~ 13.9 cm^2^ V^−1^ s^−1^) and on–off ratio (10^8^) through Mo(CO)_6_ and diethyl sulfide (DES). The DES serves as an inhibitor, which could assist the atomic layer deposition (ALD). Based on first-principles calculations of density functional theory (DFT), this can be attributed to the fact that iALD technology changes the nucleation kinetics of the Mo-containing precursor through reaction energy and steric hindrance of inhibitor molecules, thereby expanding the size of crystal grains and substrate coverage of the film.

#### Brief Sum-up

The thickness and uniformity of MoS_2_ film obtained by sulfuration reaction depend on the content and uniformity of pre-deposited precursor film, but the controllability of this process needs to be improved, and due to the restricted diffusion and inefficient sulfurization of the substrate surface, the resulting MoS_2_ film is prone to emerge large numbers of grain boundaries with relatively small size (from tens of nm to sub-μm) and irregular morphology. The solution to these problems requires further exploration in the future.

### Metal–Organic Chemical Vapor Deposition (MOCVD) Technology

**Fig. 3:**
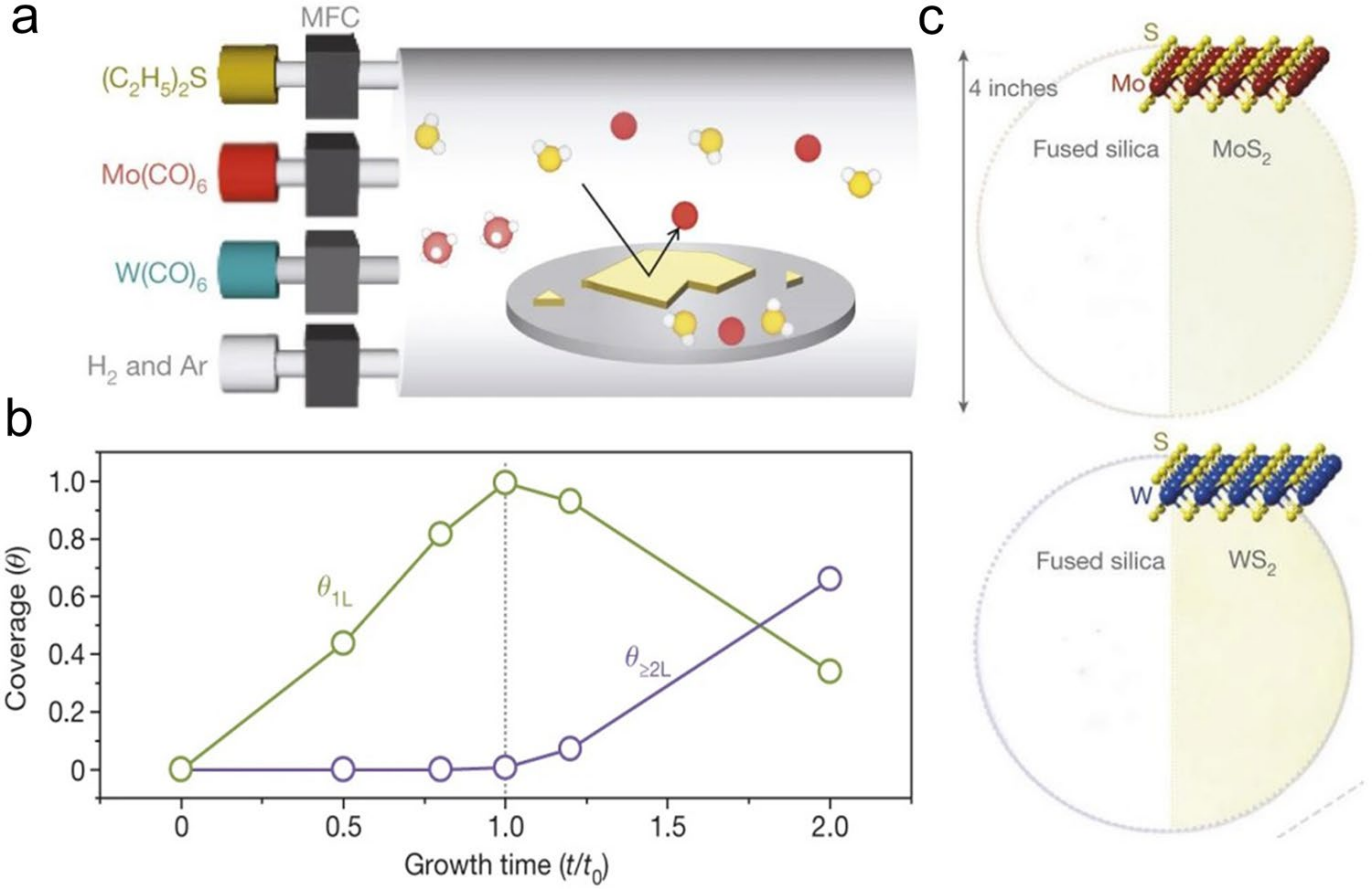
Growth the MoS_2_, WS_2_ by using MOCVD technology. **a** Diagram of the MOCVD system, **b** the coverage as the function of growth time, **c** the optical image of the 4-inch MoS_2_, WS_2_ [47], Copyright 2015, Springer Nature

Metal–Organic Chemical Vapor Deposition (MOCVD) is a non-equilibrium growth technology that was applied to the preparation of III–V (or II-VI) compound semiconductor film at an early stage and is an emerging technology of realizing wafer-scale electronic and optoelectronic materials and related heterostructures with high quality [38, 45]. MOCVD uses high-purity metal–organic compounds as a precursor, in which a huge driving force generated by pyrolysis can prepare many 2D TMDCs materials that can hardly be achieved through other technologies. Another significant advantage of MOCVD is that the growth substrate usually has no limit because large free energy variation allows single crystal-like 2D TMDCs materials to grow on various substrates without grain boundaries. Regarding size expansion and quality improvement, one effective method for MOCVD is to add salts like NaCl to inhibit nucleation, thereby increasing the crystal domain size. In addition, MOCVD can also adjust the concentration of each reactant by precisely controlling their partial pressure during growth to obtain a film with uniform electrical properties [46]. At this time, the growth of film is controlled by precursor supply rather than the interaction between precursor and substrate, which belongs to kinetics. Therefore, it is suitable for any combination of film composition and substrate, providing a way for the mass production of various 2D TMDCs materials and precise control of film thickness. Kang et al*.* reported the method of directly growing 4-inch wafer-scale single-crystal MoS_2_ film and WS_2_ film on an insulating SiO_2_/Si substrate, as shown in Fig. 3 [47]. The obtained film has good spatial uniformity and electrical properties, whose carrier mobility at room temperature is about 30 cm^2^ V^−1^ s^−1^, and can further reach 114 cm^2^ V^−1^ s^−1^ at 90 K. Cun et al*.* adopted an improved MOCVD method using sodium molybdate (Na_2_MoO_4_) and diethyl sulfide (DES, (C_2_H_5_)_2_S) as Mo source and S source, respectively, and obtained high-quality single-layer MoS_2_ film on both SiO_2_/Si substrate and sapphire [48]. The experimentally measured carrier mobility is about 22 cm^2^ V^−1^ s^−1^, showing its excellent electrical properties. Like MOCVD, this method is also not limited to the type of substrate, and DES is non-toxic, which can ensure the safety of the experiment. In MOCVD technology, the thickness and uniformity of MoS_2_ are affected by the precursor's evaporation rate and mass transfer rate. Kumar et al*.* demonstrated that when the growth temperature is higher than 850 ℃, MoS_2_ can grow layer by layer under normal pressure, and the carbon pollution caused by the organic ligand of the precursor is eliminated [49]. It is worth noting that alkali-metal-halide-assisted MOCVD technology has low applicability to low temperature and low-pressure conditions. Otherwise, a large amount of salt will evaporate or decompose before growth, which can weaken the catalytic effect. Regarding the control of morphology, Jin et al. reported a MOCVD technique for the conformal growth of atomic-level MoS_2_ film on the surface of the periodic 3D textured substrate [50]. This method used Mo(CO)_6_ and (C_2_H_5_)_2_S as Mo source and S source, and the resulting continuous film has a size of 4 inches. By fabricating a field-effect transistor based on it, the carrier mobility of the single-layer MoS_2_ was measured to be about 4.5 cm^2^ V^−1^ s^−1^. Compared with solid-phase precursors, molybdenum carbonyl hexacarbonyl is volatile, so the auxiliary effect of heating is not necessary, thereby improving the controllability and reproducibility of the MoS_2_ growth process. Kwondo et al. adopted molybdenum hexacarbonyl (Mo(CO)_6_) and dimethyl disulfide (CH_3_S_2_CH_3_) as a precursor and acquired a continuous amorphous MoS_2_ film [28]. It is notable that most organic precursors have high activity. If used together with metal oxide, the precursors are likely to be poisoned. Therefore, in CVD systems that introduce organic precursors, researchers tend to transport Mo source and S source, respectively, through different supply channels to ensure the stability and sustainability of the growth process [51], which can achieve wafer-scale (about 9.5 × 4.5 cm^2^) single-layer MoS_2_ successfully. Besides the growth of intrinsic MoS_2_ film, due to the availability of the metal vapor source, it was also able to grow doped film, such as Nb-doped MoS_2_, by using modified MOCVD. The doped concentration can be as large as 5 at%, and the Fermi level would downshift by 1.7 eV [52, 53].

Although MOCVD provides a novel way for the preparation of wafer-scale 2D materials [54], the resulting film always has many structural defects (vacancies, gaps, grain boundaries), which result in limitations in practical use and subsequent quality characterization. Besides, part of studies has shown that Mo(CO)_6_ has high toxicity [28, 38, 45], and the adsorption rate of organic compounds on the substrate surface is low. Therefore, it usually takes a long time or even a whole day to complete a wafer-level MoS_2_ film. At present, the toxicity of hydrides and the possibility of S-containing alloys appearing at ≤ 450 ℃ are found to be two other shortcomings of it. Therefore, a universal MOCVD technology has yet to be explored. The modification and technical improvement of MOCVD equipment are of positive significance for achieving stricter device specifications and higher device complexity.

### Chemical Vapor Deposition (CVD) Technology

Chemical vapor deposition (CVD) has tremendous potential for low-cost and large-scale preparation of 2D materials. It is currently one of the most promising technologies for synthesizing large-area single-crystal film and has aroused extensive research interest in the field of 2D materials. Generally, in the CVD process, the source materials should be the gas statement. However, due to the high toxicity and high corrosion, H_2_S gas has rarely been employed as the sulfur source. Similarly, metal, like Mo, based gas is rare. Thus the called CVD process during the TMDCs growth is actually a modified CVD process, where both the sulfur and metal source are supported through pre-sublimation of sulfur powder et al. or metal-based oxide/chloride et al*.*, respectively. Therefore, the CVD process for the growth of TMDCs mainly includes the following steps [55–57]:

i. Sublimation: the precursor sublimates and is transported downstream by the carrier gas
ii. Transport: the vapor diffuses toward the substrate
iii. Adsorption: the vapor is adsorbed on the substrate surface
iv. Diffusion: adsorbed atoms diffuse along the substrate surface
v. Reaction: different types of adsorbed atoms react and assemble to form films

Since the gaseous precursor of metal Mo is unstable, it is more common to adopt a solid compound as the precursor, and its vapor pressure is controllable through heating, which is a solution for obtaining Mo gases. For the S-containing precursor, due to the high activity of H_2_S, it is not only easy to react with the growth substrate but also with the reaction chamber, so its controllability is poor. In contrast, heating sulfur powder such as sublimed sulfur can obtain sustained and stable sulfur vapor, thereby realizing chemical vapor deposition of MoS_2_. The one-step CVD method is affected by all kinetic and thermodynamic factors, including precursor, substrate, etc. Therefore, it can be optimized from many aspects to achieve the preparation of high-quality, large-scale, and single-layer MoS_2_ film [58]. In the following, we will discuss the development of each factor in detail, including the precursor.

#### Mo Precursor

##### MoO_3_ Precursor

The chemical reaction equation for the conversion from MoO_3_ to MoS_2_ in Ar atmosphere is as follows [59]:

$$2{\text{MoO}}_{3} + 7{\text{S}} \to 2{\text{MoS}}_{2} + 3{\text{SO}}_{2}$$

Solid MoO_3_ powder has the potential to realize single-layer continuous MoS_2_ film at the centimeter level. However, the vapor pressure of MoO_3_ is low, and heating assistance is required to promote evaporation [60]. Besides, it is difficult to control the gas-phase combination and deposition rate, so the controllability of nucleation density, film thickness, and substrate coverage needs to be improved [49]. By adopting this method, the domain size of the MoS_2_ single crystal prepared is distributed in 10–100 μm, and carrier mobility also varies widely in 0.04–42 cm^2^ V^−1^ s^−1^. Up to now, the domain size of single-crystal MoS_2_ prepared by this method can be as large as 100 μm. On the other hand, further study has pointed out that as a non-quantitative suboxide of MoO_3_, MoO_3-x_ possesses higher reactivity and can better react with S vapor or Se vapor to produce MoS_2_ or MoSe_2_ [61–63]. Taking MoSe_2_ growth, for example, Li et al*.* proposed a three-step growth model by analyzing the quenched samples when MoO_3_ reacting with S atoms was not completed and proved that the growth of MoSe_2_ is a reversible process, which was supported by the fact that the edge of the MoSe_2_ film recedes during slow cooling. At the same time, they also pinpointed that the metastable nanoparticle reaction intermediate (such as MoO_0.79_Se_0.24_) can be used as a direct source of Mo source and nucleation sites growth [64].

##### MoO_2_ Precursor

**Fig. 4:**
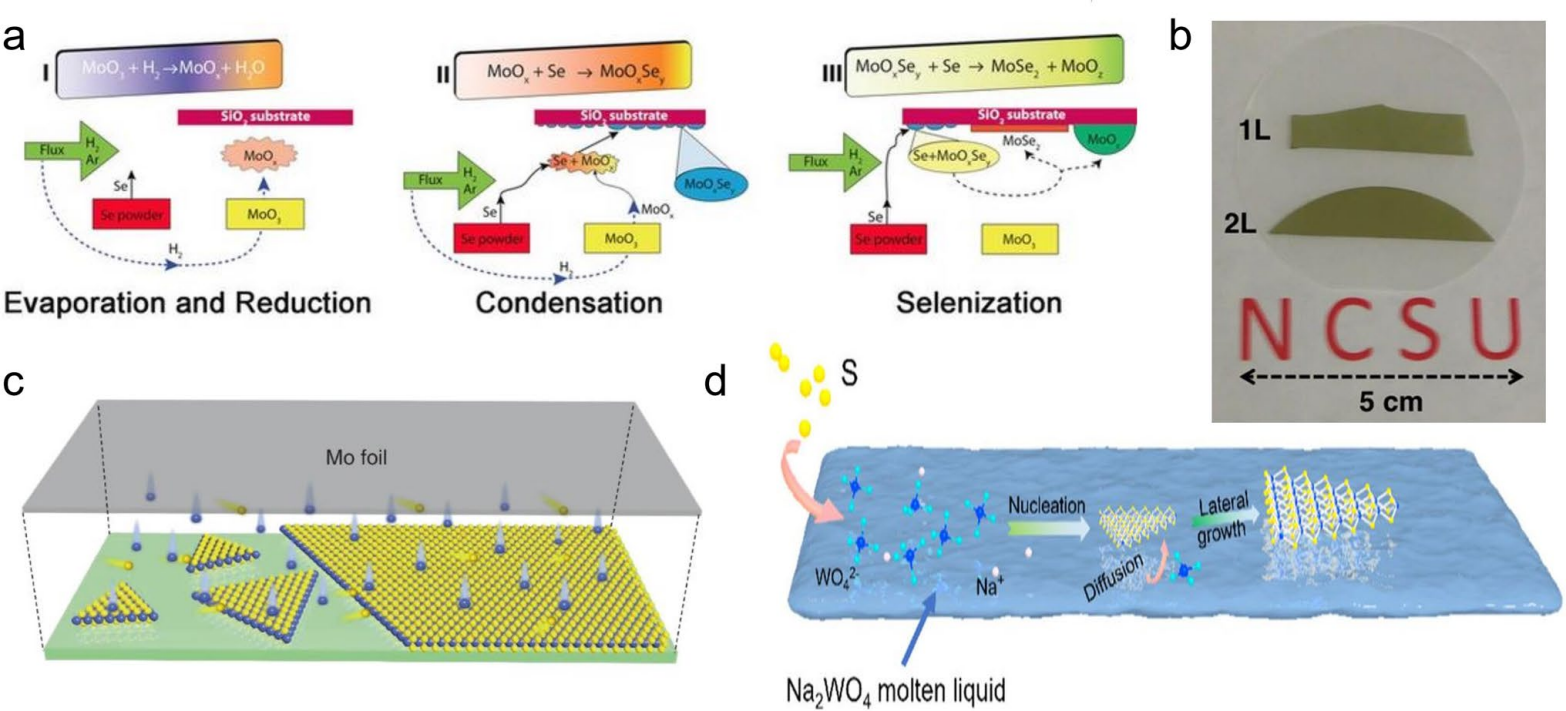
The outcome of film influenced by precursor type. Growth mechanism by using **a** MoO_3_ as the precursor [64], Copyright 2016, WILEY–VCH. **b** MoCl_5_ as the precursor [65], Copyright 2013, Springer Nature. **c** Mo foil as the precursor [66], Copyright 2018, Springer Nature. **d** Na_2_WO_4_ as the precursor [67], Copyright 2020, American Chemical Society

MoO_2_ microcrystals possess the potential to grow MoS_2_ film with high crystallinity and strictly controllable layers. This can be attributed to the orderly accumulation of Mo atoms on its surface, which ensures that after replacing O atoms with S atoms, the resulting MoS_2_ layer remains a crystalline structure. Based on MoO_2_ microcrystal, Wang et al*.* reported the growth of MoS_2_ single crystal with a domain size of 10 μm. Limited by the diffusion rate, the number of sulfuration layers within MoO_2_ crystallite, that is, the thickness of MoS_2_ film is controlled by sulfuration time, as shown in Fig. 4b. Further electrical tests showed that the device performance of the back-gated field effect transistor (FET) based on the obtained film is comparable to that of the FET based on MoS_2_ mechanically exfoliated, which is proof of its high crystallinity [30].

##### Mo Foil

Mo foil can provide a new supply method, face-to-face, thereby avoiding the inherent weakness of density attenuation in point-to-face source supply. However, it should be noted that the pure Mo foil cannot be used as the Mo source during the growth because the vaporization temperature of Mo foil is too high. Instead, researchers found that the pre-oxide Mo foil is suitable for growth due to the formed Mo oxide on the Mo foil. The plane structure of the Mo oxide facilitates the formation of the uniform Mo-based precursor gas [68]. Zheng et al*.* first oxidized the Mo foil by electrochemical oxidation and then covered the growth substrate forming a confined space. It was found that the as-grown MoS_2_ flakes have gradually shrunk basal planes and deliver high carrier mobility as significant as 55 cm^2^ V^−1^ s^−1^. The shrinking basal planes provided more transport channels resulting in small contact resistance [68]. Yang et al*.* adopted Mo foil as the Mo-containing precursor to efficiently realize the growth of MoS_2_ film with a domain size of 400 μm within 8 min, and the film showed excellent optical and catalytic properties, crystal quality, and uniformity as well, as shown in Fig. 4c [66]. Comparing the two supply modes of point-to-face and face-to-face, it was found that the latest fashion mainly improves the film thickness uniformity by reducing the concentration gradient fluctuation of Mo-containing precursor in the gas phase during the transmission process. MoO_3-x_ produced after Mo foil oxidation can fill the gap between substrate and Mo foil uniformly so as to achieve uniform nucleation and continuous growth. However, in the case of point-to-face, the concentration decline of MoO_3_ is unavoidable when transported downstream by carrier gas, thus limiting the domain size.

##### MoCl_5_

MoCl_5_ can be easily evaporated into gas to support the growth of MoS_2_ film, and has also been directly acting as a Mo-containing precursor, but it is toxic to humans [69, 70]. However, the evaporated temperature is much lower at about 200–300 °C. Thus it is hardly controlled. Although it can grow MoS_2_ on a large scale, the large ratio of the MoCl_5_ leads to the fast growth of MoS_2_ and results in the amount of the grain boundary [65]. Therefore, its electrical property is usually a little bit low. Yu et al*.* demonstrated the growth of large-scale MoS_2_ by using MoCl_5_ and found that the carrier mobility was 0.003–0.03 cm^2^ V^−1^ s^−1^ [65]. Browning et al*.* used MoCl_5_ and H_2_S as precursors of atomic layer deposition (ALD) to obtain double-layer MoS_2_ film, but its carrier mobility only reaches 1 cm^2^ V^−1^ s^−1^, which is far lower than the applicable standard of microelectronics/nanoelectronic devices [71]. Therefore, the growth method using MoCl_5_ to grow MoS_2_ directly needs to be further explored to improve the film quality and preparation safety.

##### (NH_4_)_2_MoO_4_

Dissolving soluble compound in a solvent and spin-coating it on the substrate as Mo source is a deformation of face-to-face supply, which avoids not only the reduction of domain size due to high nucleation density but also the shrinking of size limit caused by concentration fluctuations. Alkali metal salts of transition metal acid have the characteristics of low melting point and easy solubility in water, which can achieve uniform dispersion of the precursor when spin-coated on the substrate. And then, reactants melt, migrate and react with S atoms during the heating process. Recently, Yun et al*.* reported spin-coated AMT [(NH_4_)_6_H_2_W_12_O_40_·x(H_2_O)] on the sapphire substrate as a W source and placed it on the opposite side of the Au foil (growth substrate), which was able to achieve single crystal MoS_2_ film with the size of 420 μm and continuous film of 2 × 3 cm^2^ [66, 72]. Qin et al*.* demonstrated that the liquid-assisted CVD method introduces a gas–liquid-solid growth mode, which enables the resulting film with higher crystalline quality and size [73]. They used an aqueous solution of Na_2_MoO_4_ as a precursor, which was firstly spin-coated on SiO_2_/Si substrate, and then heated in a sulfur atmosphere. By a similar method, uniform WS_2_ film with a domain size of up to 100 μm can be obtained using Na_2_WO_4_ as the precursor. Liu et al*.* found that molten intermediate compounds could induce the growth of a uniform continuous film [67]. This can be attributed to the fact that the migration barrier of liquid is lower than that of solid. Therefore, it can effectively avoid unnecessary aggregation of reactive atoms. In addition, liquid-assisted-CVD can also induce a self-limiting growth mechanism during growth. That is, the film can stop vertical growth at a specific number of layers, which is of positive significance for researchers wanting to obtain single-layer MoS_2_ films in most cases. Through this method, Liu et al*.* reported the growth of large-scale monolayer continuous WS_2_ film [67]. The size of the domain reaches 110 μm, and the transistor based on continuous WS_2_ film showed extraordinary performance, whose on/off ratio and carrier mobility are 10^8^ and 10 cm^2^ V^−1^ s^−1^, respectively. Similarly, Yan et al. successfully synthesized monolayer and bilayer MoS_2_ films by spin-coating H_8_MoN_2_O_4_ aqueous solution on a substrate [74]. The prepared MoS_2_ photodetectors showed excellent photoelectric characteristics, whose responsivity and detection could respectively reach up to 7160 A W^−1^ and 2.44 × 10^11^ Jones, exceeding many other types of photodetectors. Their research also indicates that the liquid precursor can control the thickness of the resulting film through the concentration of H_8_MoN_2_O_4_. For example, the researcher found that the as-grown MoS_2_ would evolve from monolayer to bilayer or multi-layer when the solution increased from 4 to 6 μL.

In summary, the advantages of alkali metal salts of transition metal acids are twofold: i) It ensures the uniform distribution of Na_2_WO_4_ particles, then the growth and merging of multiple crystals contribute to the formation of continuous films; ii) When heated at high temperatures, particles on substrate melt into liquid phase, which is easier to migrate to the nucleation sites. And this helps the expansion of a single crystal nucleus. The advantages of the solution-assisted CVD method are gradually being explored. It provides a facile approach for efficiently synthesizing large-scale, high-quality two-dimensional materials, including MoS_2_. It is also a good way to achieve uniform doping and other heterostructures by adjusting the composition of liquid-phase sources, such as Nb-MoS_2_ and V-WSe_2_ [73, 74].

#### Sulfur Precursor

There is the rare sulfur precursor. Although some organic sulfide might be used in MoS_2_ growth, the potential carbon contamination limits its application. Therefore, only sulfur powder, H_2_S, (NH_4_)_2_MoS_4_, and CS_2_ have been used as the sulfur precursor. Recently, Robertson et al. reported that sulfur could also be provided by annealing the MoS_2_ itself, and the sulfur vapor is more stable [29]. In the following, we will discuss the influence of the sulfur precursor.

##### S Powder

S powder is currently the most widely used S source. Its advantages, such as low toxicity and high vapor pressure, and the ability to combine with various Mo sources to generate MoS_2_ add the possibility for its popularization in a laboratory. Najmaei et al. used MoO_3_ and S powder as precursors and obtained a MoS_2_ film with carrier mobility of about 4.3 cm^2^ V^−1^ s^−1^ and an on/off ratio of 6 × 10^6^. Zhan et al*.* heated S powder to 750 °C and sulfurized Mo metal film to form single-layer MoS_2_ films with a size of about 0.8 × 0.8 cm^2^. The carrier mobility of about 0.04 cm^2^ V^−1^ s^−1^ is possible due to its small grain size. Although S powder is the earliest and most used source of S, its inherent drawbacks of uncontrollability and inhomogeneity have always plagued people. This can be attributed to the non-uniformity of sublimation and diffusion when heated, known as the position-dependent phenomenon. Therefore, many articles on finding alternative S sources and improving supply sources have been reported.

##### H_2_S

H_2_S has a strong sulfurization ability and can be better controlled than other compounds, providing a stable sulfur source. Kim et al*.* employed H_2_S as a precursor to directly grow polycrystalline monolayer MoS_2_ film with a size of 5 × 2 cm^2^ on a SiO_2_/Si substrate [51]. However, when H_2_S is used together with metal oxides (WO_3_, MoO_3_, etc.), it is easy to lead precursor poisoned. Thus the metal oxides are hardly evaporated. Separating sulfur and molybdenum sources in two quartz tubes and introducing to near the growth substrate can effectively avoid such issues [51]. Liu et al*.* synthesized a wafer-scale (9.5 × 4.5 cm^2^) continuous MoS_2_ film on the sapphire substrate through a dual-channel CVD system, which avoided the problem of oxide precursor premature sulfurization [51]. In addition, the remaining metal oxide precursor can be recycled after annealing, significantly reducing costs. In addition, introducing O_2_ can also effectively solve the same problem. Chen et al. adopted an O_2_-assisted CVD method to improve the film quality and synthesized monolayer monocrystalline MoS_2_ film with a size of 10 μm [51]. Although H_2_S is one of the most effective S sources and can effectively suppress C-doping, some safety problems still exist. First, H_2_S is a highly toxic flammable compound, and its acceptable concentration limit for humans is only 20 ppm. H_2_S would significantly prolong the reaction time compared with S powder, and it takes as much as 26 h to form a single-layer MoS_2_. Therefore, adopting the CVD method for preparing high-quality MoS_2_ film by H_2_S needs to be further explored.

##### (NH_4_)_2_MoS_4_

(NH_4_)_2_MoS_4_ can provide both S source and Mo source simultaneously, mainly used for liquid-phase-assisted sulfuration reactions. The homogeneous solution, including MoS_2_, WS_2_, or MoWS_2_ alloy, ensures film uniformity [75, 76]. In the conversion process, ammonium thiomolybdate ((NH_4_)_2_MoS_4_) firstly undergoes pyrolysis in the N_2_ atmosphere, that is, it is converted to MoS_3_ in the temperature range of 120–360 °C. Secondly, as further increasing the temperature (> 800 °C), MoS_3_ is converted to MoS_2_. One-step conversion can be achieved in the above process by replacing Ar with H. The reaction formula is as follows [31]:

$$\left( {{\text{NH}}_{4} } \right)_{2} {\text{MoS}}_{4} \to 2{\text{NH}}_{3} + {\text{H}}_{2} {\text{S}} + {\text{MoS}}_{3}$$

$${\text{MoS}}_{3} \to {\text{MoS}}_{2} + {\text{S}}$$

$$({\text{NH}}_{4} )_{2} {\text{MoS}}_{4} + {\text{H}}_{2} \to 2{\text{NH}}_{3} + 2{\text{H}}_{2} {\text{S}} + {\text{MoS}}_{2}$$

The advantage of (NH_4_)_2_MoS_4_ is that it provides both sulfur and molybdenum, reducing the complexity of the reacting system. However, the limited sulfur source causes the film to have numerous sulfur vacancies, and the crystal domain of the film is as small as tens of nanometers. Although large-scale molybdenum disulfide film can be prepared quickly in this method, the crystal quality remains to be further improved.

##### CS_2_

The decomposition of CS_2_ in hot molybdenum wire was found to support the growth of wafer-scale MoS_2_ film, and the film thickness is strictly controlled. During this process, after the molybdenum wire is heated, the generated volatile MoS_x_ can be directly deposited on a substrate and can be controlled in a monolayer by colorimetry [77]. Almeida et al. used CS_2_ as S source to successfully realize wafer-scale MoS_2_ film [78]. The growth process does not depend on a specific airflow distribution, and the properties of resulting MoS_2_ are superior to mechanical-exfoliated MoS_2_ and CVD-MoS_2_ in terms of current noise characteristics. Further studies have shown that controlling the growth temperature can also contain the volatile substances with correct element composition. Considering CS_2_ can support the sulfur source, recently, it has also been used to post-treat the TMDCs film, including MoS_2_ [79]. It was found that the carrier mobility of the CS_2_-treated MoS_2_ is in the range between 0.2 to 0.6 cm^2^ V^−1^ s^−1^, which is much large than that of none treated MoS_2_, with mobility in the range between 0.005 and 0.01 cm^2^ V^−1^ s^−1^.

#### Ratio of Precursors

**Fig. 5:**
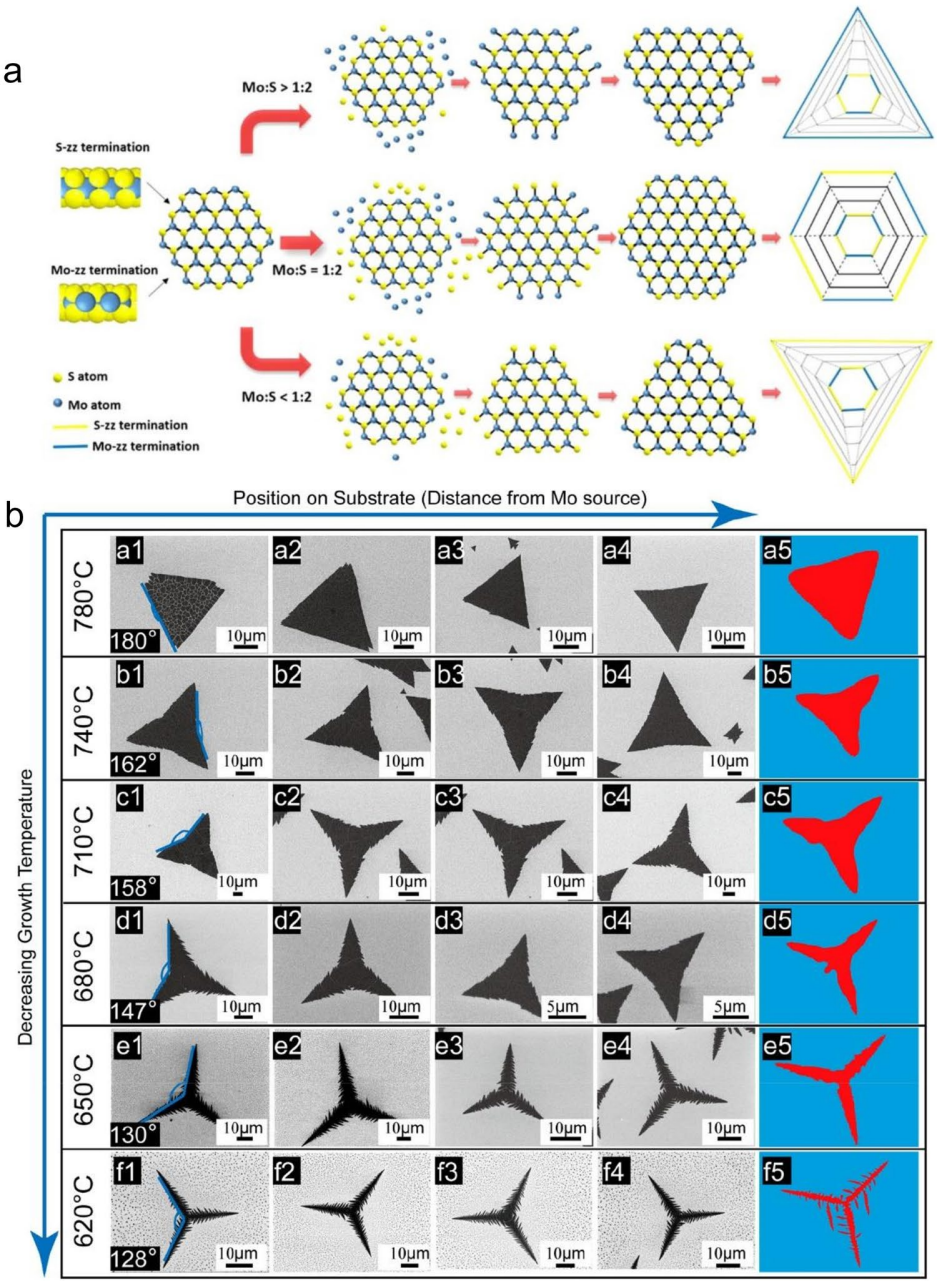
The MoS_2_ shape as the function of the growth condition. **a** Influence of Mo:S ratio [60], Copyright 2014, American Chemical Society. **b** Influence of growth temperature and substrate position [81], Copyright 2021, American Chemical Society

It is reported that flux fluctuations in the ratio of Mo source to S source can affect the chemical composition of the terminal edge in MoS_2_, thereby changing the morphology of the film, as shown in Fig. 5a [60]. This can be attributed to the non-equilibrium growth process at the crystal edge. The S and Mo edges are zigzag edges, but the two edges have different growth kinetics [80]. The S atoms exposed on edge only form 2 bonds with Mo atoms (3 in the saturated state). Only 4 bonds are formed with S atoms (6 in the saturated state) for the Mo atoms exposed on edge. The difference in structure causes them to exhibit different chemical activities under different ratios. Very recently, Xu et al*.* reported that the MoS_2_ shape significantly depends on the growth conditions, which would modulate the adatom concentration profile property resulting in different growth mechanisms, as shown in Fig. 5b [81]. The researchers realized the MoS_2_ shape range from triangles, concave triangles, and three-point stars to dendrites.

According to the ratio of precursors, it can be divided into three types of Mo:S (> 1:2, 1:2, and < 1:2) [38, 60, 82–86]: in the first case, Mo is sufficient, the S-zz end grows faster than the Mo-zz end, the edge with unsaturated S atoms is exposed to the air, causing it more unstable in energy than Mo edge and more likely to meet with Mo atoms and form a bond. Finally, the film forms a triangular shape and ends with a Mo edge. Under the second condition, Mo:S ratio corresponds to the stoichiometric ratio of MoS_2_. The stability of the two ends and the probability of meeting the other free atoms are similar, resulting in similar growth rates. The shape of the film is generally hexagonal. The third case is identical to the first case, the film is triangular, but the end is S edge. It is worth noting that the ratio of Mo atoms to S atoms on the substrate surface affects the energy stability of Mo and S edges and may lead to twin-defect-derived growth, both of which will result in changes in the shape of the crystal.

In addition to the difference in morphology, a suitable precursor flux ratio can also suppress 60°-oriented domains and achieve the purpose of unifying crystal orientation. Experimental results show that when the S: MoO_3_ ratio is relatively low (~ 2:1), the number of MoS_2_ domains with 0° and 60° orientations is equivalent, similar to the epitaxially grown MoS_2_ on mica. When S: MoO_3_ ratio is close to 3:1, the proportion of 0° domains gradually increased from ~ 49% to 64% and finally to 98%. Previous articles proved that growing the MoS_2_ on h-BN by molecular beam epitaxy can inhibit the formation of reverse 60° MoS_2_ by introducing ultra-low Mo flux. Thus, it can be seen that lower Mo flux is conducive to MoS_2_ growing in a thermodynamically balanced state, which is independent of the substrate.

Regarding the mechanism by which how ratio of precursors regulates the orientation of the MoS_2_ domain, Aljarb et al*.* proposed that the orientation of the initial MoS_2_ seed determines the orientation of MoS_2_ [87]. Additionally, it affects domain orientation by affecting the size of the MoS_2_ seed. This can be attributed to the fact that small-sized nuclear is more likely to rotate to an energy advantage, that is, 0° domains, so it is much easier for the substrate to unify the orientation of the small-sized nuclear and lose control of large-sized ones. It is worth noting that the base lattice determines the energy advantage. Therefore, to obtain large-area single-crystal MoS_2_, a high S: MoO_3_ ratio should be maintained in the initial nucleation stage to promote the formation of MoS_2_ seeds. During the later stage of size expansion, it should be reduced to increase the adsorption of reacting atoms for growth improvement. The recent results also showed that excessively high S partial pressure could significantly increase the nucleation rate of MoS_2_ but accelerate the nucleation in the vertical direction simultaneously, and the rate in vertical is faster than in horizontal, resulting in multi-layer MoS_2_ particles [83]. One can tell from the above phenomena that when S partial pressure is controlled around an appropriate value, we can achieve the best combination of lateral nucleation rate and growth rate to realize a large-scale single-layer MoS_2_ pattern.

Regarding the control of precursors ratio, it can be directly controlled by conditions such as temperature and pressure or indirectly affected by factors such as source spacing and carrier gas flow rate. Özden et al. achieved a reduction in the number of layers of MoS_2_ by adjusting the distance between the substrate and MoO_3_ powder (gradually increasing from 2.5 to 11.5 cm), and found that the flux ratio corresponding to 4.5 cm is the growth window for single-layer MoS_2_ sheet [88]. And the range of 5.5 ~ 9.5 cm is suitable for single-layer MoS_2_ growth, corresponding to the S:MoO_3_ ratio of 66–150. Guo et al. found that the variation of the Mo:S ratio can be realized by adjusting the entry time of the S source, further controlling the degree of sulfuration in precursor film (MoS_2_ thickness) [83]. This is essentially a modification of Mo and S partial pressure. Studies have shown that [S]/[Mo] is negatively correlated with the entry time of S. When S is introduced in an early stage, [S]/[Mo] is high enough to completely sulfide MoO_3_ into MoS_2_. When S is introduced later, [S]/[Mo] decreases rapidly, resulting in only a part of MoO_3_ transfer into MoS_2_. If S enters much later, then only black MoO_2_ particles can be obtained.

#### Growth Atmosphere

**Fig. 6:**
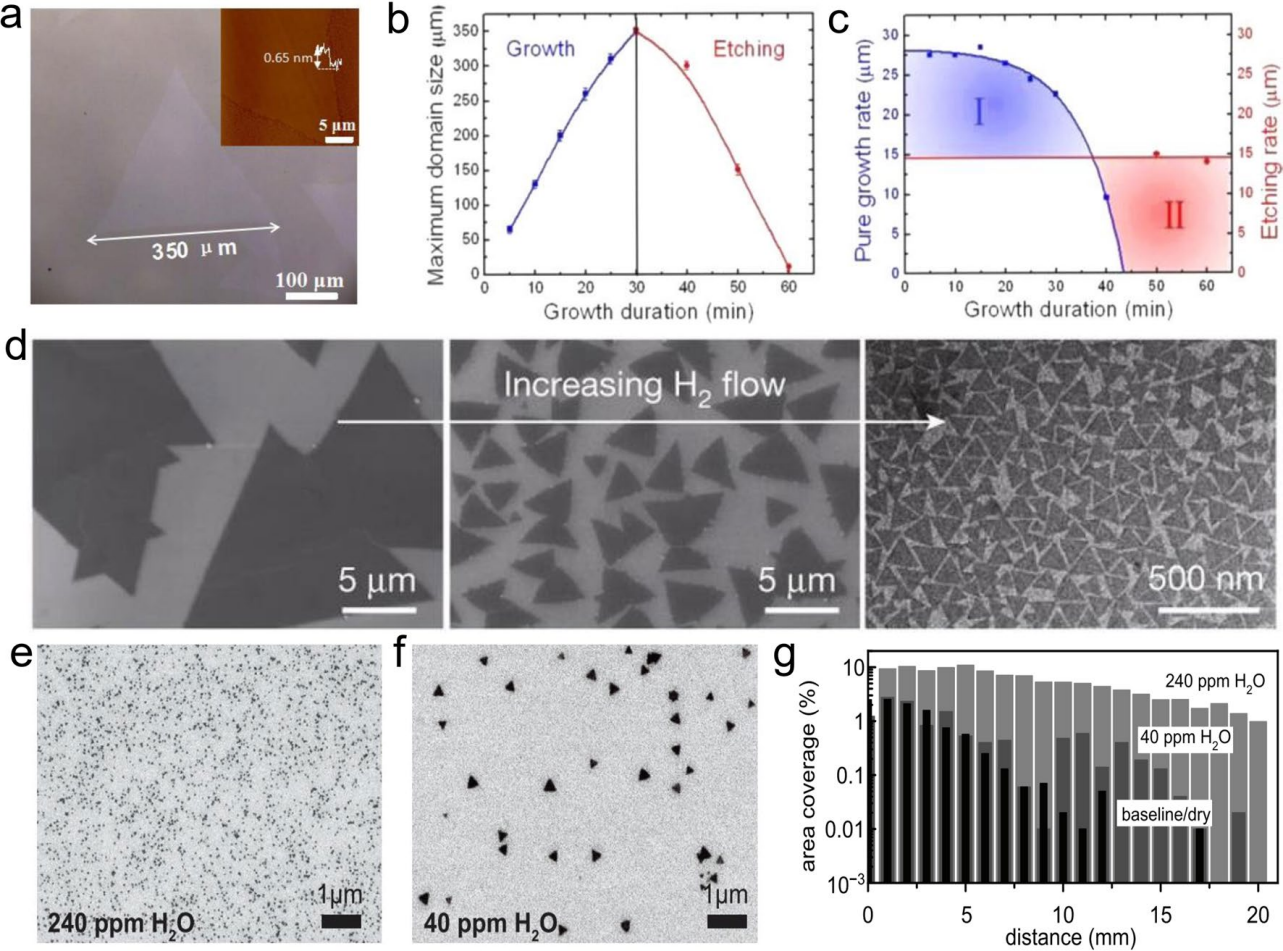
The effect of the used atmosphere. **a**–**c** Using oxygen mixed Ar as the atmosphere [90], Copyright 2015, American Chemical Society. **d** Using H_2_ mixed Ar as the atmosphere [47], Copyright 2015, Springer Nature. **e**–**g** Using H_2_O mixed Ar as the atmosphere [91], Copyright 2017, Institute of Physics

The growth atmosphere also plays an essential role during the growth. Ar and N_2_ inert gas is commonly employed as the carrier gas which realizes mass transport. Recently, it was found that some mixture gas, such as oxygen or hydrogen, would tune the growth mechanism, including the growth speed, nuclear density, etc., as shown in Fig. 6. Besides the used gas, the pressure of the atmosphere also significantly affects growth.

##### Atmosphere Type

As an inert gas, Ar gas is the most commonly used carrier gas. Since it does not participate in any reaction during the growth process, it only dilutes and transports the reactant, so it has almost no effect on MoS_2_ growth. However, a suitable carrier gas may solve the unsolvable problems of precursor, substrate, temperature, etc. Recently, it was discovered that some gases could promote the growth of MoS_2_ and etch unnecessary nuclei, such as O_2_, and H_2_. Therefore, more and more researchers tend to mix them with Ar gas to promote film growth. Like Ar, N_2_ is an inert gas and does not participate in reactions. Van der Zande et al*.* only used N_2_ as the carrier gas and obtained monolayer single-crystal MoS_2_ film with high quality, showing comparable photoelectric properties to that of mechanically exfoliated MoS_2_ [89]. And the maximum domain size reached 120 μm. The carrier mobility is about 8 cm^2^ V^−1^ s^−1^.

Among carrier gases, pure Ar does not participate in reactions. But other reactive substances, such as H_2_, impact the morphology of MX_2_ film. H_2_ can act as a reducing agent to promote the reduction of WO_3_ powder [18]. Also, it can create a WO_3-X_ rich environment directly or indirectly through the formation of H_2_S, transforming the growth model from diffusion-limited growth to attachment-limited growth and promoting the generation of a thermodynamically stable triangular film. Takenobu et al*.* and Huang et al*.* found that H_2_ can activate the reaction of WO_3_ and Se to obtain WSe_2_ monolayer film: WO_3_ + 3Se + H_2_ = WSe_2_ + H_2_O + SeO_2_ [32, 59]. Similarly, Sial et al*.* successfully expanded the domain size of MoSe_2_ to 60 μm and even formed a continuous film with the assistance of H_2_ [56].

$${\text{MoO}}_{3} + 3{\text{H}}_{2} + 2{\text{Se}} \to {\text{MoSe}}_{2} + 3{\text{H}}_{2} {\text{O}}$$

Compared with MoSe_2_ film, it is optional to introduce H_2_ or other reducing agents during the growth of MoS_2_ film. Because the reactivity of Se atoms is much lower than that of S atoms, a potent reducing agent is needed to help the selenization process of WO_3_. The thermodynamic calculations of WO_3_ selenization are only consistent with the condition existing of H_2_. However, H_2_ can promote the reduction of metal oxide powder, its effect peaks with the change in content. When the content of H_2_ is relatively high, the average grain size increases significantly. On the contrary, the morphology is more regular. Further studies have shown that appropriate H_2_ content can improve the grain structure, average grain size, and intercrystalline connection of MoS_2_ films [46]. Similarly, in preparation for MoSe_2_, Gong et al. obtained continuous MoSe_2_ film with a domain size of 1 mm through a tunable CVD technique and found that it is impossible to synthesize MoSe_2_ without H_2_. In the presence of H_2_, film thickness and defect density are simultaneously affected by growth temperature and H_2_ flux [86]. When the H_2_ ratio is low (≤ 7.5%), the single-layer MoSe_2_ film contains triangular holes, and the bottom film of the double-layer MoSe_2_ film is continuous. Still, the top film has defects, which the decomposition of MoSe_2_ may cause. On the other hand, the research of Chen et al*.* showed that increasing the concentration of H_2_ to a specific value (> 30%) will enhance the H_2_ etching effect on MoSe_2_ [92]. They also found that when the proportion of H_2_ is too high, WO_3_ is rapidly reduced to metal W. At this time, the evaporation rate of WO_3_ slows down, which is not conducive to the formation of WSe_2_ film. Further experimental results show that the optimal ratio of Ar/H_2_ mixed gas is about 4:1. In addition, according to the experiments of Liu et al*.*, when the H_2_ concentration is excessively high, its etching effect similar to that of O_2_ can be exhibited, resulting in smaller lateral dimensions and more grain boundary [67]. This also proves that suitable H_2_ content is required to obtain a high-quality film. As a reactant, H_2_ can also react with the dangling bonds on the substrate surface to change its chemical properties.

Recently, during growing 2D-TMDCs in a CVD system, it was found that the presence of O_2_ can significantly inhibit the density of MoS_2_ crystal nuclei, maintain the reactivity of precursors, eliminate growth defects, and expand domain size. This can be attributed to the etching effect caused by O_2_. That is, O_2_ can chemically oxidize the edges, etching unstable nuclei. Chen et al*.* synthesized high-quality single-crystal monolayer MoS_2_ film using O_2_-assisted CVD technology [51, 90]. It was found that the size of the MoS_2_ domain is significantly dependent on the flux of the oxygen, where it can grow to as large as 350 μm when the oxygen flux is 2 sccm, as shown in Fig. 6a [90]. However, when the oxygen flux rises to 5 sccm, the size would go down to 50 μm. The introduced oxygen has been proposed to both prevent the poisoning of the MoO_3_ and eliminate defects during the growth. The obtained MoS_2_ has a carrier mobility of 90 cm^2^ V^−1^ s^−1^ at room temperature. Inspired by this novel phenomenon, Lan et al*.* found that the critical role of O_2_ in WS_2_ growth changes with its concentration [93]. When the O_2_ concentration is 0, the WS_2_ domain size is only 30 μm, and when the O_2_ concentration is 0.5%, the domain size expands to 80 μm, and when the O_2_ concentration is 1.0%, the average domain size can reach 210 μm. However, when the O_2_ concentration continues to increase beyond 1.0%, the excess O_2_ shows an entirely different effect, that is, etching:

$${\text{O}}_{2} + {\text{WS}}_{2} \to {\text{WO}}_{{\text{x}}} + {\text{SO}}_{{\text{y}}}$$

Although O_2_ has a specific auxiliary effect on MoS_2_ growth, O_2_ is not a prerequisite for the growth of large-scale crystals and is also dangerous for people. Therefore, the safety of O_2_-assisted CVD technology needs to be further improved.

##### Growth Pressure

Yu et al*.* proved that the partial pressure of gas-phase MoS_2_, P_Mo_, can control the deposition of MoS_2_ films [65]. Because the difference between the partial pressure P_Mo_ and the vapor pressure P^o^_Mo_ provides the thermodynamic driving force for the reaction, the conditions for the reaction to proceed are P_Mo_ > P^o^_Mo_. According to the law of mass reaction, the partial pressure P_Mo_ determines the deposition rate, so a higher P_Mo_ corresponds to a faster deposition rate. Further studies have shown that the thermodynamic balance between P_Mo_ and P^o^_Mo_ may induce a self-limiting growth mechanism. By precisely controlling P_Mo_ between the P^o^_Mo_ of single-layer and double-layer films, the film growth can be automatically stopped at a specific number of layers, thereby hindering the multilayer growth. At this time, the number of film layers is only determined by the chamber pressure and has nothing to do with the continuous supply of gas-phase MoS_2_. In addition, Jung et al. confirmed that the two growth modes of hexagonal multilayer and triangular double-layer growth could be controlled by regulating the gas flux and pressure [56]. Yang et al*.* demonstrated that the size of the MoS_2_ can be tuned by both the growth temperature and the pressure [20]. It was found that the MoS_2_ domain can be grown as large as 400 µm when the pressure is about 3 kPa. Further increasing the pressure, the MoS_2_ size would decrease.

Atmospheric pressure chemical vapor deposition (APCVD) has the potential to expand the crystal MoS_2_ domain [60, 86]. van der Zande et al*.* managed to adopt APCVD technology to obtain triangular MoS_2_ film with a domain size of about 120 μm [18]. Chen et al*.* separated the induction stage and growth stage in APCVD, reducing nucleation density and obtaining MoS_2_ with a domain size of 305 µm [15]. The obtained film has high crystallinity, and electrical properties, whose carrier mobility and on–off ratio reach 30 cm^2^ V^−1^ s^−1^ and 10^6^, separately. Through improvement, He et al*.* adopted APCVD to realize the layer-by-layer growth of TMDCs films utilizing partial feeding [94]. The continuous film size is about 4.7 × 6 cm^2^, showing higher flatness and consistent orientation. In addition to the advantages of size expansion and simple process [95]. Gao et al*.* found that single-layer WS_2_ film grown on Au foil by APCVD has a weak interaction force with the substrate, which is conducive to subsequent film transfer [96]. However, the interaction between the film prepared by low-pressure chemical vapor deposition (LPCVD) and the substrate is strong, making it hard to achieve non-destructive electrochemical bubbling. Wang et al*.* found that APCVD can improve the crystalline quality of NbSe_2_ film, which can further act as a superconducting material [97]. Through APCVD, they obtained single-layer NbSe_2_ films with a domain size of over 200 μm and a deficient defect concentration. In short, APCVD technology is not limited to film composition and substrate type and can be widely applied in different combinations of film and substrate.

LPCVD can improve the quality of materials through a large mean free path of the vapor precursor [94, 95]. Similar to APCVD, it is widely used in the growth process of 2D-TMDC materials because of its widely applicable process. In existing reports on LPCVD technology, the preparation of MoS_2_ film is mainly based on sulfurization Mo or MoO_X_, but the thickness of the obtained film could be better controllable. Later, researchers replaced the Mo source with volatile MoCl_5_ [94], and obtained a uniform minority (1–3 layers) film by adjusting the partial pressure. However, the crystal domain was still limited, which in turn affected the carrier mobility. Recently, atomic-thickness WS_2_ film has been realized by LPCVD technology [18]. A low-pressure environment can accelerate the evaporation and diffusion of WO_3-X_, while inhibiting the desorption of WO_3-X_/WS_2_ clusters, ensuring nucleation uniformity. As mentioned above, LPCVD is more complicated than the APCVD process, but APCVD may sacrifice film purity [96]. In an experiment of growing MoS_2_ film by APCVD technology on Au foil, Mo has high solubility and is easy to form Mo-Au surface alloy with the substrate. And it is difficult to control the number of film layers through environmental pressure [96]. Therefore, LPCVD technology can reduce the supply of Mo and S sources to reduce the occurrence of such phenomena.

Besides the used gas and the pressure, it was reported that the gas flux and flow direction also affect the growth. Zhang et al*.* proposed a film growth process that controls the supply of precursors by countercurrent to avoid unnecessary nucleation, thereby promoting rapid nucleation and size expansion under optimal growth conditions [98]. It can be explicitly expressed in the heating stage. The carrier gas reverses to inhibit unnecessary nucleation before reaching the growth temperature. As soon as reaching the optimal growth temperature range, it stops switching, and the precursor supply is sufficient at this time, which is conducive to rapid nucleation. When the temperature is further increased, the collection of gas-phase reactants and the mobility of the adsorbed atoms on the substrate surface rise simultaneously. At this time, the nucleation process is completed and begins to expand the film size. The carrier gas flow rate mainly affects the nucleation density and film morphology by controlling the transport of precursor. When the carrier gas flux is low, the transport of precursors on the substrate surface is difficult, while most of the precursors will be blown away under high carrier gas flux conditions. Therefore, the appropriate carrier gas flow rate can optimize precursor concentration, facilitating the growth of large-scale MoS_2_ film. Based on the research of Sial et al*.*, it was found that low flow rates tend to form large-area monolayer film due to low nucleation density, and high flow rates tend to cause small-sized crystal domains due to increased nucleation density [56].

#### Catalyst

##### Alkali Metal Halide

**Fig. 7:**
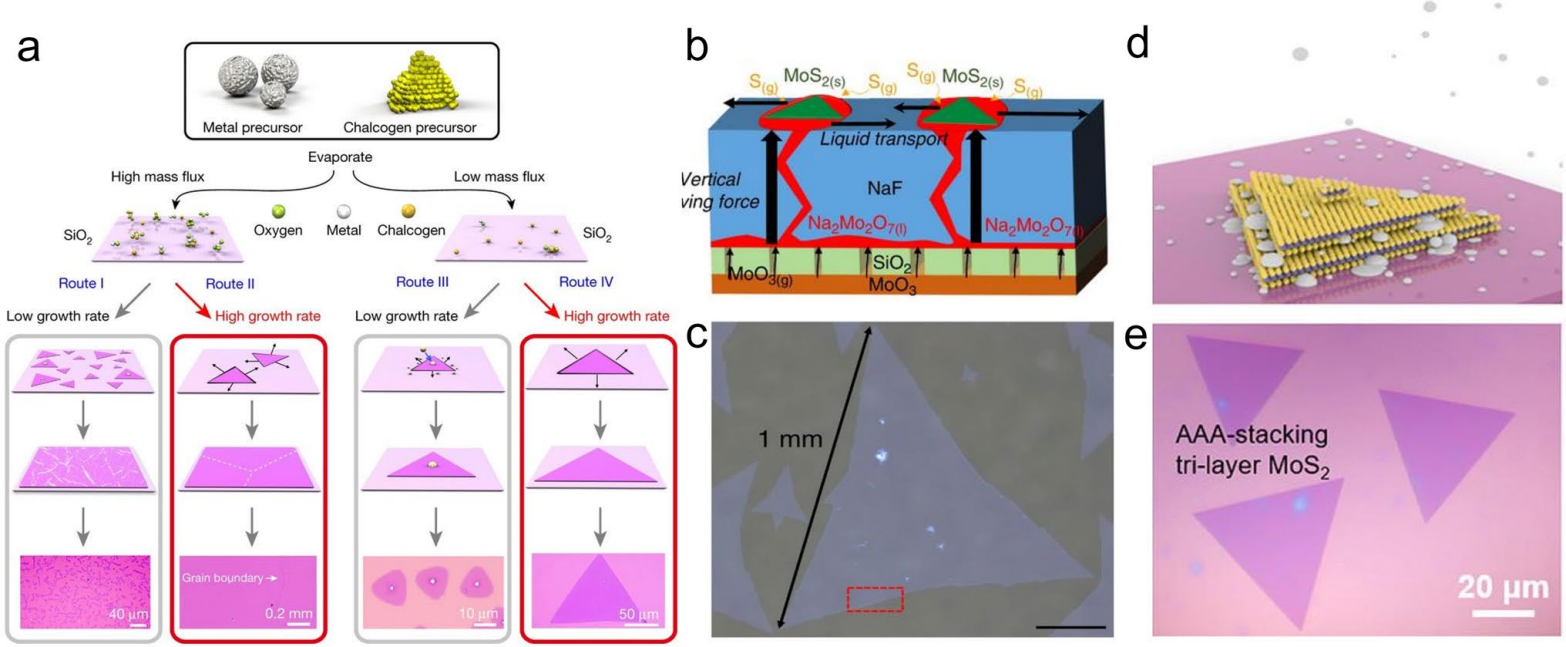
Catalytic growth of TMDCs by alkali metal halides. **a** Catalytic mechanism [100], Copyright 2018, Springer Nature. **b**, **c** The realization growth of 1 mm-size MoS_2_ [74], Copyright 2020, Springer Nature. **d**, **e** Growth of AAA-stacking tri-layer MoS_2_ [101], Copyright 2021, American Chemical Society

Alkali metal halides have been found to act as a catalyst to support the transport of metal precursors and to expand film by inhibiting nucleation, including NaCl, KCl, KI, etc. [99, 100]. Taking NaCl, for example, a small amount of NaCl can lower reaction temperature and reduce energy consumption. It can be applied to both liquid and solid phase methods. In the former case, NaCl and the metal precursor are configured into a homogeneous solution at first, which then is spin-coated on the substrate; this can promote the migration of adsorbed atoms during the heating to support the rapid growth of wafer-scale MoS_2_ film, as shown in Fig. 7a. The influence of NaCl in expanding film size is distinct. Kang et al*.* compared the results of NaCl assisted or not MoS_2_ formation process and found that NaCl or KCl can increase the film size to two orders of magnitude [45]. Chang et al*.* demonstrated that single crystal MoS_2_ film with a domain size of 450 μm through the self-capping vapor–liquid-solid method (SCVLS), as shown in Fig. 7b, c [74]. And the quality of the obtained film is outstanding, with a carrier mobility of 49 cm^2^ V^−1^ s^−1^ and an on–off ratio of ~ 5 × 10^8^. Recently, as shown in Fig. 7c, Luo et al*.* demonstrated that introducing NaCl would activate the basal plane of MoS_2_ and facilitate its multilayer growth, such as AAA-stacking tri-layer MoS_2_ film [101].

Similarly, in the growth of other types of transition metal chalcogenide film, Lan et al. reported a NaCl-assisted method to increase the W supersaturation in a semi-sealed quartz tube effectively. Thereby, the size of the expanded WS_2_ reaches 1.7 mm [31, 102]. Wang et al*.* proposed that the growth of single-layer NbSe_2_ superconductor film does not necessarily require an ultra-high vacuum environment (different from MBE) [97], but alkali metal halide is a must. This can be attributed to the fact that the melting point of niobium oxide is generally higher than 1510 °C, and it is difficult to spontaneously evaporate into the gas phase and react with Se vapor. While molten alkali metal halides can form intermediate products with metal oxides, some studies have shown that they are metal oxychlorides, which have lower melting points than before. Li et al*.* proposed that NaCl can expand the size of a monolayer single-crystal MoSe_2_ film to 250 μm while maintaining the average carrier mobility of 12 cm^2^ V^−1^ s^−1^ [46]. The improvement effects of NaCl in MoSe_2_ growth are mainly reflected in the following two points: i) MoO_2_Cl_2_ is formed and then reacts with Se and H_2_ in the gas phase; ii) The melting point of MoO_3_ is reduced, which effectively increases the mass flux of the metal precursor.

Recently, it was found NaBr can mediate the growth of monolayer TMDCs film [95]. NaBr does not react with MoO_2_, therefore, does not cause the precursor to liquefy. The researchers used the pre-deposited NaBr pattern as a template to achieve the growth of a monolayer large-scale MoS_2_ sheet with different patterns, solving the size limitation problem and simplifying the subsequent device manufacturing process. It was found that alkali metal halides can effectively improve film purity. Traditional CVD methods will inevitably introduce C impurities, and the presence of amorphous C will change the film properties from semiconductor to semi-metal. Therefore, the introduction of NaCl has a positive meaning for maintaining its semiconductor properties. Song et al*.* obtained MoS_2_ film with carrier mobility of 10.4 cm^2^ V^−1^ s^−1^ and an on–off ratio of 3 × 10^7^ by introducing volatile S-containing organic compound and NaCl [103]. The optical microscopy, Raman spectroscopy, X-ray photoelectron spectroscopy, photoluminescence, and transmission electron microscopy measurements all support that NaCl-assisted CVD-MoS_2_ film has large grain size, clear Raman shift, strong photoluminescence, good Stoichiometric ratio, and sixfold coordination symmetry. To further discuss the catalytic mechanism inside NaCl, Song et al*.* found that the catalytic effect of NaCl on the synthesis of MoS_2_ is based on Na_2_S_x_ chains formed at the MoS_2_ grain edge. When the temperature reaches 700 °C, NaCl evaporates, adsorbs on the substrate's surface, and then reacts with DMS. At the same time, H_2_ can remove CH_3_ ligands, which is conducive to the generation of Na_2_S_x_. Finally, Na_2_S_x_ diffuses and moves to the MoS_2_ grain edge, forming the Na_2_S_x_ chain (x > 1). The reaction between the Na_2_S_x_ chain and MoCl_5_ can be expressed as follows:

$${\text{MoCl}}_{5} + \frac{5}{2}{\text{Na}}_{{\text{s}}} {\text{S}} \to {\text{MoS}}_{2} + 5{\text{NaCl}} + \frac{1}{2}{\text{S}}$$

Although alkali metal halides have advantages in increasing continuous MoS_2_ film size, some disadvantages exist. Zhang et al. proposed that NaCl can induce the substrate surface to be saturated with Na–O and form the Na–O interface to inhibit charge transfer, thereby limiting the application of TMDCs [45]. In addition, NaCl can increase the nucleation density. If the merging of the adjacent film cannot be achieved, it may lead to a polycrystalline film [102]. In addition, alkali metal halides may also form by-products under TMDCs materials, such as Na/K metal oxides [38].

##### NaOH and KOH

In the process of growing graphene by a CVD method, the existence of O_2_ can form -OH, thereby reducing the energy of H [104]. According to the Bell-Evans-Polanyi principle, the activation energy of edge dehydrogenation is correspondingly reduced, thereby promoting the growth of single-crystal graphene on a large scale. In addition, O_2_ can also catalyze the decomposition of hydrocarbons and increase the supply and edge adhesion of C atoms [105]. Xu et al*.* obtained circular single-crystal graphene with a lateral size of 0.3 mm in only 5 s in the existence of O_2_ [106]. Compared with the absence of O_2_, the growth rate was several orders of magnitude faster. Belonging to 2D materials such as graphene, MoS_2_ can be catalyzed by -OH. Zhu et al. found that the -OH group can promote the number of single-layer MoS_2_ film and achieve 3 × 3 cm^2^ single-layer MoS_2_ continuous film on sapphire through a -OH bilayer-mediated method while having carrier mobility as high as 30 cm^2^ V^−1^ s^−1^ [107]. This can be attributed to the preferential connection of -OH groups to the surface of MoS_2_ (001), forming a MoS_2_-OH double-layer structure and introducing the S-Mo-S-OH growth mode [96, 107]. The S-Mo-S-OH growth mode can hinder MoS_2_ from growing vertically along the [001] crystal axis, thereby limiting its growth direction to horizontal. In addition, -OH can repair S defects (predicated by DFT) and effectively prevent MoS_2_ from interfacial oxidation in the air, which is beneficial to stabilize its electrical properties. The study of Lan et al. showed that NaOH could increase the flux of the W source in the gas phase and react with WO_3_ on the surface of the W foil to transform into Na_2_WO_3_ [93], thereby promoting the growth of large-scale WS_2_ sheet:

$${\text{NaOH}} + {\text{W}} + {\text{O}}_{2} \to {\text{Na}}_{2} {\text{WO}}_{3} + {\text{H}}_{2} {\text{O}}$$

And

$${\text{Na}}_{2} {\text{WO}}_{3} + {\text{S}} \to {\text{Na}}_{2} {\text{O}} + {\text{WS}}_{2}$$

This can be attributed to the higher volatility of Na_2_WO_3_, which makes it easier to transport downstream and react with S vapor.

#### Growth Substrate

**Fig. 8:**
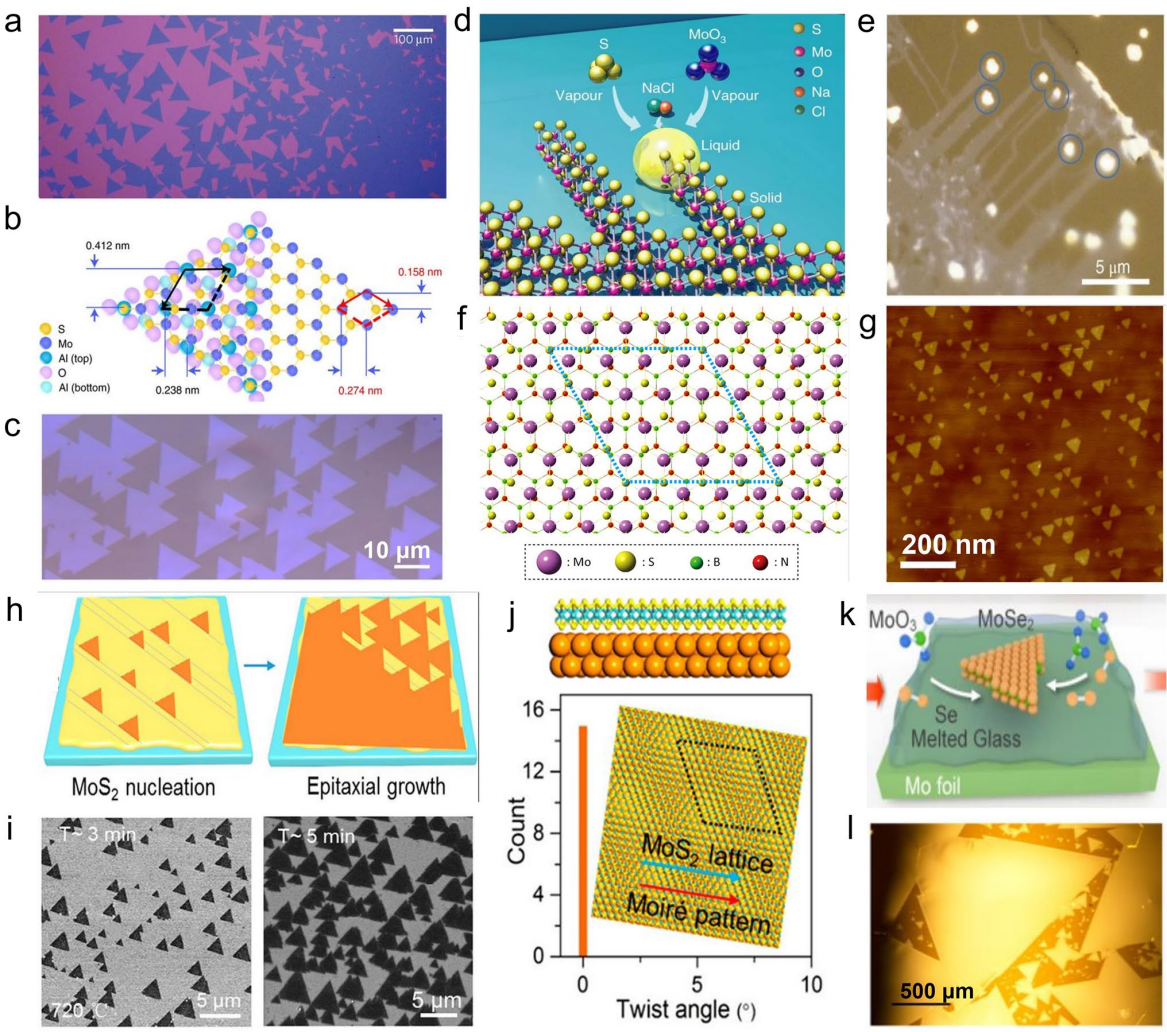
Influence of growth substrate on the MoS_2_ film. **a** Random distribution and orientation of MoS_2_ on amorphous SiO_2_ surface [89], Copyright 2013, Springer Nature. **b**, **c** Epitaxially growth of MoS_2_ on sapphire substrate [108], Copyright 2021, Springer Nature. **d**, **e** Vapor–liquid-solid growth of MoS_2_ nanoribbon on NaCl substrate [109], Copyright 2018, Springer Nature. **f**, **g** Epitaxially growth of MoS_2_ on hBN substrate [110], Copyright 2017, American Chemical Society. **h**–**j** Epitaxially growth of MoS_2_ on Au substrate [84], **k**, **l** growth of MoSe_2_ on melted glass [92], Copyright 2017, American Chemical Society

Substrates can be divided into two types: crystalline and amorphous. Crystalline substrates have different lattice parameters, so the morphology of films grown on other substrates varies accordingly, as shown in Fig. 8. Meng et al*.* controlled the morphology of MoSe_2_ film employing mastering the characteristics of different substrates [32]. Wan et al*.* found that the morphology of MoS_2_ film is affected by the adsorption energy and diffusion energy barrier of the gas-phase reactant on a substrate surface [85]. Higher total adsorption energy and lower diffusion energy barrier help the adsorbed atoms stay longer on a substrate, move longer distances, and are more conducive to the growth of the high-quality, large-scale 2D film. By comparing a variety of substrates, the sapphire substrate has advantages in preparing single-layer continuous MoS_2_ film due to its excellent absolute adsorption and diffusion energy. The two types of energy corresponding to the graphene substrate are relatively low, so it is easy to form a high-density multi-edge MoS_2_ single crystal. In contrast, SiO_2_/Si substrate is unfavorable for the growth of both single-crystal and polycrystalline film, which may be caused by lower total adsorption energy or diffusion energy barrier. The choice of the substrate should be based on the specific film composition [56]. Otherwise, the lattice mismatch will inevitably lead to a generation of non-uniform tensile strain, thereby creating various structural defects and decreasing the film's quality.

##### Solid Substrate

Solid substrates include SiO_2_/Si, h-BN, mica, glass, sapphire, and other insulating materials and metals. Except for Au, most metals react easily with Mo to form alloys or with S to form metallic sulfide. However, the high price of Au foil limits its application in the laboratory. Insulating single crystal substrate has uniform surface atoms, high chemical inertness (low O doping level), and few charge traps [18, 85, 111, 112], which can be used to obtain high-quality single-layer MX_2_ materials. As mentioned above, the SiO_2_/Si substrates and sapphire substrates commonly used in laboratories do not have higher adsorption energy and lower diffusion energy barrier at the same time, thereby cannot adapt to the preparation of large-scale single crystal film. Therefore, insulating substrates require modifications to suit the direct growth of MoS_2_ [58, 83].

**SiO**_**2**_ **/Si substrate**

The compatibility of SiO_2_/Si substrate with modern electronic products makes it an ideal substrate for growing large-scale single-crystal MoS_2_ film [15]. The electrical properties of MoS_2_ on SiO_2_/Si substrate are mainly limited by domain size [18]. Researchers have made many attempts to expand further the application of MoS_2_ film in the field of new microelectronic devices. Lu et al*.* obtained a single-layer MoSe_2_ continuous film of 1 × 1 cm^2^, using SiO_2_/Si substrate as a growth substrate [113]. Zhan et al*.* used SiO_2_/Si substrate as a growth substrate and broke the limitation of growth conditions on the film size through sulfide Mo film [33]. In the transfer process, the hydrophilic SiO_2_/Si substrate greatly simplifies its difficulty. Cheng et al*.* pre-treated SiO_2_/Si substrate with plasma, and the results showed that the treated rough area effectively reduced nucleation density and increased film growth rate [114]. Through further calculation, the growth rate of the rough area can be 3 times that of the polished area.

**Sapphire substrate**

*C*-plane sapphire has the same lattice symmetry as MoS_2_, and can be used for the epitaxial growth of single-crystal MoS_2_ film. First principles calculation shows that high charge density and binding energy of WS_2_/sapphire interface are conducive to the lateral growth of film, thereby realizing the expansion of film size. Aljarb et al*.* demonstrated that sapphire surfaces could be reconstructed to form long terraces and wide steps at high temperatures, thereby controlling film morphology [87]. Huang et al. adopted sapphire as a growth substrate and obtained triangular single-crystal WSe_2_ film at 850 °C, with a maximum lateral size of up to 50 μm [59]. Lan et al. obtained single-layer WS_2_ film with a domain size of up to 800 μm by optimizing growth conditions and found that the morphology of film induced by sapphire was mostly single-layer and triangle [102]. This can be attributed to the stronger bond coupling between WS_2_ and sapphire shown by the DFT calculation, manifested in the decrease of charge interaction distance. In addition, WS_2_ film has a higher binding energy (− 182.4 meV) on the sapphire, which can reduce the energy barrier during lateral growth together with the bonding state. In addition to morphological differences, sapphire may also affect crystal orientation. Ji et al. found that monolayer triangular MoS_2_ crystals grown on sapphire substrate mainly have two orientations, 0° and 30° [111]. Further imaging of the grain boundaries shows that adjacent MoS_2_ crystal domains generally exhibit an armchair crystal orientation. Although sapphire is an ideal substrate for growing 2D materials such as MoS_2_ [59], it is expensive, so there is an urgent need to reduce the process cost of sapphire or find alternative substrates.

**Quartz substrate**

The main component of quartz is SiO_2_. There are few reports on quartz as a growth substrate, and most of them show that it has a negative effect on the large-area growth of MoS_2_ films. For example, Yin et al. used quartz as a growth substrate and obtained MoS_2_ and WS_2_ films with regular triangular or hexagonal shapes [115]. But the AFM test showed that the uniformity of the obtained films was poor. This can be attributed to the fact that quartz, as an amorphous solid substrate, inevitably has impurities and defects on its surface, which can cause an accumulation of reacting atoms, leading to irregular grain boundaries and uneven thickness. In addition, the lattice mismatch between quartz and MoS_2_ will aggravate the defect density at the interface, resulting in further deterioration of crystal uniformity, which can be identified by the increase of FWHM in the Raman spectrum.

**Mica substrate**

Muscovite mica is a flexible layered material with high surface energy and hydrophilicity, so it can serve as a flexible substrate with a strong adsorption capacity. AFM shows that the surface of muscovite mica has atomic-level flatness, and so do the TMDCs films grown on it. Bao et al*.* obtained flexible transparent photodetectors by loading MoS_2_ film with inherent mechanical flexibility, strong light trapping ability, and high carrier mobility on muscovite mica [116]. Under 660 nm laser irradiation, the photodetector's responsivity and detection can reach 8.45 µA W^−1^ and 4.1 × 10^7^ Jones, respectively. The thermal stability conferred by muscovite mica makes it stand out among other types of photodetectors, showing extraordinary application potential. This can be attributed to the fact that the heat distortion temperature of muscovite mica (> 300 °C) is much higher than that of other flexible substrates, such as PET, PEEK, and PDMS (~ 200 °C). Similarly, Liu et al*.* used muscovite as a substrate to obtain a SnTe film and fabricated a flexible near-infrared (NIR) photodetector [117]. Under the irradiation of a laser with a wavelength of 980 nm, it exhibited significant photoelectric properties, with its detection and responsivity reaching 3.89 × 10^8^ Jones and 698 mA W^−1^, respectively.

In addition, the film grown on muscovite mica can be oriented preferentially, showing the characteristics of van der Waals epitaxy. In-plane XRD test showed that SnS_2_ with [2110] or [1210] orientation was oriented along the mica [98] direction. The difference between the second preferred direction and the first preferred direction is 60°, which the mirror symmetry of the muscovite surface may cause. Mattinen et al*.* obtained a small number of continuous SnS_2_ films with an area of 5 × 5 cm^2^ through low-temperature atomic layer deposition technology and further expanded their size substrate by Van De Waals epitaxy through annealing on the mica [118]. It was proposed that by optimizing the growth conditions, the resulting SnS_2_ morphology can be adjusted to triangular crystallites or continuous films [118]. By further comparing the effects of different substrates on SnS_2_ during the annealing process, Mattinen et al. concluded that mica is superior to sapphire and SiO_2_/Si substrates [118]. It can enhance the out-of-plane texture and reduce the roughness of film at 300 ℃, while these phenomena were not observed on the other two substrates and side reactions occurred instead, forming Sn_2_S_3_ or SnS. It is worth noting that although the lattice constants of SnS_2_ and muscovite mica are quite different, epitaxy can still be achieved through coincidence site lattice or domain matching epitaxy concept.

**Metal substrate**

Graphene growth can choose Cu-Ni alloy as a substrate to change C diffusion mode from surface diffusion to quasi 3D diffusion, thereby improving the solubility of C [119–121]. At the same time, the growth mechanism also changes from surface-mediated growth to isothermal segregation, and the growth rate increase significantly. Li et al. replaced the rigid substrate with a flexible Cu foil and obtained highly uniform graphene film with a lateral dimension of up to 30 inches [122]. One way to obtain large-scale single-crystal film is to control the evolution of a crystal nucleus into a large-sized film. Therefore, Wu et al*.* obtained single-crystal graphene up to 1.5 inches in size through local precursor feeding [106]. Some studies have shown that folding, annealing, and electrochemical polishing of metal substrates can effectively suppress nucleation density. Similar to the growth of graphene, the metal substrate has identical effects on the growth of 2D transition metal chalcogenide films. However, the high activity of the Cu substrate would react with sulfur, thus making it unable to grow MoS_2_. Yun et al*.* realized a single crystal WS_2_ film with a domain size of 420 μm using chemically inert Au foil [72]. Shi et al*.* obtained high crystalline quality, domain size adjustable (from 200 nm to 50 μm), and strictly monolayer MoS_2_ film [123]. They also developed the isolated crystal into a continuous one with an area of about 3 × 3 cm^2^ on Au foil through LPCVD technology. The reduction reaction of MoO_3-x_ on Au foil can be expressed as:

$${\text{S}}_{2} + {\text{MoO}}_{{3 - {\text{X}}}} \to {\text{MoS}}_{2} + {\text{SO}}_{2}$$

It is worth noting that MoS_2_ film grown on Au foil has a good electrocatalytic activity for hydrogen evolution reaction, which shows a low Tafel slope (61 mV dec^−1^) and higher exchange current density (38.1 μA cm^−2^), which can be attributed to the strong electronic coupling between film and Au foil. Another method to obtain large-sized single-crystal film is to use a lattice-matched substrate through a multi-nucleation approach to unify domain orientation and merge them into a continuous single-crystal film. It has been reported that single-layer monocrystalline hexagonal boron nitride can be epitaxially grown on the adjacent surface of Cu (110). The resulting h-BN crystal domains achieve a single orientation by coupling the zigzag edge of h-BN with Cu (211) step edge. Therefore, the step edge of the Cu (111) surface can be used to induce single orientation growth of h-BN. Similarly, Au (111) surface perfectly matches the lattice symmetry of MoS_2_, which can be used as a growth template to synthesize MoS_2_ film with low defect density [124]. Yang et al. used Au (111) single crystal as a substrate and obtained a 1-inch MoS_2_ continuous film through APCVD technology. In addition to the size expansion, the metal substrate also allows lossless transfer. In another study, Gao et al. reported that WS_2_ film induced by Au substrate has a weak interaction force with the substrate and can be transferred to any substrate by electrochemical bubbling, avoiding the corrosion of strong acid and alkali [96]. Moreover, the peeled Au foil can continue to be used, reducing the experimental cost to a certain extent.

Although metal substrate is an option, as mentioned earlier, most metals tend to form compounds or alloys with precursors and interfere with film growth. Only a few specific 2D materials do not sacrifice purity, such as WS_2_. Because Au can reduce the barrier of sulfuration of WO_3_ [93], it can ensure the WS_2_ film continues emerging at a lower concentration of WO_3_ and S.

**Graphene-layered materials**

MoS_2_ film induced on graphene substrate mostly exhibit 60° symmetry, implying the strict epitaxial feature of MoS_2_ on graphene [85, 125]. Also, h-BN has similar epitaxial properties for MoS_2_ growth. Since the substrate surface significantly impacts the quality of TMDCs materials, Wan et al*.* fabricated graphene (Gr)-SiO_2_ interface and Gr-Al_2_O_3_ interface to modify local free energy and then used them as a substrate for growing film. The results show that different types of interfaces exhibit additional adsorption energy and diffusion energy barriers to precursors, affecting the MoS_2_ morphology. Zhou et al*.* reported the growth of PtSe_2_ on the MoSe_2_, where the PtSe_2_ domains have the epitaxy growth mechanism on the MoSe_2_ surface [126].

##### Liquid Substrate

Multilayer film grown on a solid substrate surface has been proven to be thermodynamically stable, so it is difficult to suppress the generation of multilayer MoS_2_ film with a solid substrate. This can be attributed to the difficulty in controlling the supply of Mo-containing precursors.

**Molten Glass**

Glass with the super hydrophilic property has also been used to grow TMDCs, whose growth temperature is very close to the melting point of the glass. The slight melting surface of the glass facilitates the nuclear of TMDCs and improves its self-assembly to form a film with large grain size. Özküçük et al*.* proposed that the reason why the glass has a catalytic effect is due to the Na^+^ inside, not the glass surface [127]. Therefore, the glass can only act as a catalyst during the growth of the TMDCs materials, and the film should still be grown on a hydrophobic substrate. Further studies indict that glass with higher Na + content is more likely to obtain uniform MoS_2_ flakes and standardize its morphology, which is consistent with Özküçük's view. Although there is no catalytic effect, the surface of molten glass still has a lubricating effect at high temperatures, which can increase the diffusion and migration rate of adsorbed atoms. Yang et al*.* took soda-lime glass as a growth substrate, acquiring single crystal MoS_2_ with a domain size of 400 μm [66]. The carrier mobility of the MoS_2_ is in the range of 6.3 to 11.4 cm^2^ V^−1^ s^−1^, which can be further improved by optimizing the fabrication.

Soda-lime glass is low-cost and meets the economic requirements of mass production. Na^+^ in soda-lime glass has been proven to have a catalytic effect on the growth of MoS_2_ film. Sum et al*.* reported the use of molten glass to synthesize single-layer MoS_2_ continuous film with a size of 2.5 mm [107]. Due to the excellent flatness and isotropy of molten glass surface, the resulting film has advantages in defect density and growth rate. Chen et al*.* used molten glass to break through the reported size limit of TMDCs materials [92]. Subsequent studies on atomic-scale imaging and photoelectric transmission confirmed the film has high crystalline quality. Yang et al*.* successfully synthesized a single-layer MoS_2_ film with a lateral size of 400 μm using molten glass [66]. In addition, the film shows excellent optical and catalytic properties, crystal quality, uniformity, and growth rate. Compared with a quartz substrate, a glass substrate can increase film growth rate by 38 times. It is worth noting that the catalysis of glass in film growth does not originate from its smooth surface. Yang et al*.* placed a glass substrate upstream of quartz (growth substrate) and observed the experimental results. They found that the metal elements in glass are auxiliary to the growth of the film. Further comparing the effects of Na^+^ and Ca^2+^ [66, 127], it is Na^+^ that has the catalytic effect. In addition to the catalytic effect, ionic compounds in the glass can also corrode Mo foil (precursor) to promote the volatilization of the Mo source [66], thereby increasing the precursor concentration.

Although its smooth surface cannot catalyze the formation of materials, it can accelerate the rate of atom migration and transform the rate-limiting step from a mass transfer process to a surface reaction. At this time, the reaction is transformed from thermodynamic control to kinetic control, which is easy to achieve high-coverage continuous MoS_2_ film. The calculation results show that other substrates need to provide 10^5^ times the precursor to meet the requirements for continuous film formation. In addition, there is a novel substrate-trapping strategy (STS) in the liquid substrate by dissolving the overflowing Mo-containing precursor into itself to form a stable MoO_4_^2−^ in a molten state, which can greatly inhibit non-uniform diffusion and nucleation [32, 112]. When melting, the flat surface of glass facilitates the size expansion of single crystal MoS_2_. Loh et al*.* proposed using molten glass with a smooth and defect-free surface as a growth substrate to obtain triangular MoS_2_ crystals and millimeter-level continuous films with side lengths of more than 350 μm. And the carrier mobility of monolayer film is as high as 90 cm^2^ V^−1^ s^−1^.

Recently, Cai et al*.* reported a new synthesis method of two-dimensional transition metal dichalcogenides that can control the supply and diffusion of reactants, called dissolution–precipitation (DP) growth [128]. Specifically, it can be expressed as: the metal source is embedded between two pieces of glass, with the top one much thinner than the bottom one. Therefore, the supply of Mo source requires Mo atoms to diffuse and pass through the glass substrate on it and then react with S vapor when reaching the surface. At this time, Mo atom concentration on glass surface is uniform, and it is easy to obtain single-layer films. This method is similar to graphene's growth by dissolving carbon sources in bulk or sub-surface of a catalytic substrate, like nickel, and then obtaining large-scale graphene on a substrate surface. The researchers proposed that a similar mechanism can also be applied to the synthesis of TMDCs materials and can avoid the attenuation of the local concentration of precursors. Additionally, it is proved that the DP method can reduce the possibility of by-products produced during gas-phase reactions. As a result, the purity and electrical properties of the film are guaranteed, with a carrier mobility of 7.5–21.5 cm^2^ V^−1^ s^−1^ and on/off ratio of 10^6^–10^8^. In order to verify the hypothesis of improved cleanliness, Cai et al*.* launched a comparative experiment with TiCl_4_ as adsorbent (TiO_2_ particles formed by the hydrolysis of TiCl_4_ can be selectively absorbed by contaminated areas). Consistent with their expectation, the results show that MoS_2_ film obtained by DP method adsorbs the least TiO_2_ particles compared with traditional CVD-MoS_2_. In addition, PL spectrum shows the strong interlayer coupling between two adjacent vertical MoS_2_ layers in the double-layer film, which also verifies the high cleanliness of the film. Regarding uniformity, the MoS_2_ flakes they obtained were highly uniform, with an average nucleation density of up to 1080 flakes mm^−2^, and an average crystal domain size of up to 10 μm. This may be attributed to the high uniformity of reactants after diffusing, which is supported by the AFM image that the precursors (Na_2_MoO_4_, Na_2_WO_4_, NaVO_3_) sealed between two layers of glass produce many protrusions with a lateral size of about 2 μm and a height of about 20 nm when crossing the upper glass. These protrusions are then converted into MoS_2_ when they meet with S atoms.

In short, the glass substrate has many excellent characteristics, and its substrate-trapping strategy is universal, which can be further applied to TMDCs films of other components. Finally, the hydrophilicity of glass greatly reduces the pollution and fragmentation during the transfer process, making it possible to transfer without damage.

**NaCl liquid substrate**

NaCl is a kind of single crystal, and the Na + inside has been found in many articles to catalyze the large-scale growth of MoS_2_. Li et al*.* reported a method of obtaining high-quality monolayer MoS_2_ crystal ribbons through a gas–liquid-solid mode using NaCl substrate, as shown in Fig. 8d, e [109]. After many times of optimization, the width of the MoS_2_ crystal ribbon can reach thousands of nanometers. In their experiment, a layer of MoS_2_ film was first deposited on the surface of the NaCl substrate to achieve epitaxial growth of the MoS_2_ ribbon. The results proved that the pre-deposited MoS_2_ could indeed control the ribbons' orientation in the upper layer, which is supported by the fact that crystal ribbons aggregate at the lower MoS_2_ grain boundary. AFM test shows that the orientations of MoS_2_ ribbons grown upper on adjacent grains present about 120° turn. NaCl serves as both substrate and catalyst, resulting in the pre-deposited MoS_2_ layer forming Na–Mo–O droplets, and when S is saturated, MoS_2_ is grown in a "crawling mode" and finally forms into a band shape. The possible reaction formula is as follows:

$${\text{3MoO}}_{{3}} \left( {\text{s}} \right) + {\text{2NaCl}}\left( {\text{s}} \right) \to {\text{Na}}_{{2}} {\text{Mo}}_{{2}} {\text{O}}_{{7}} \left( {\text{l}} \right) + {\text{MoO}}_{{2}} {\text{Cl}}_{{2}} \left( {\text{g}} \right)$$

Through EDX analysis acquired the particle composition, it was demonstrated that the particles contain molybdenum, sodium, oxygen, sulfur, and a few chlorine [109]. When changing the growth substrate to SiO_2_/Si substrate, a similar result of emerging ribbon-shaped MoS_2_ with particles inside can also be achieved using NaCl as a catalyst. This supports the primary role of the Na_2_Mo_2_O_7_ compound instead of the substrate in impacting the films' morphology. In some areas, 2D-MoS_2_ flakes can be found without particles inside. Therefore, it was speculated that the particle itself is an intermediate and provides sources for reaction. Under certain conditions, the particles will be exhausted and disappear if the reaction proceeds entirely.

To understand the inherent dynamics of "crawling mode", Li et al*.* further studied the source of the driving force of droplet motion. They believe that the movement of the droplet depends on the relative value of the interface-free energy (σ_1_) of the droplet/crystal ribbon and the interface-free energy (σ_2_) of the droplet/substrate when σ_1_ > σ_2_, the overall interface-free energy (Gint) increases. Because σ_Gint_ = (σ_1_-σ_2_)σ, MoS_2_ nucleates and grows laterally on the droplet/substrate interface. And if MoS_2_ continues to grow, the free energy increases with the size of the crystal domains. This process is similar to the spontaneous migration of a tiny droplet from a low surface to a high-energy one. Once the droplet's movement proceeds, sulfur, and MoO_3_ will continue dissolving in it, thereby inducing the continuous growth of the film and obtaining a crystal ribbon structure. The synthesis method of this unique 1D-on-2D structure proposed by Li et al*.* provides an idea for quickly obtaining complex nanostructured TMDCs materials and their heterostructures. When applied to MoS_2_, this rapid synthesis of a large-area high-quality film can be achieved by determining an appropriate liquid phase intermediate. Similarly, Graham et al*.* reported textured photosensitive MoS_2_ with a size of approximately 1 × 1 cm^2^ through a solid-phase reaction [129]. A series of subsequent property tests showed that its electrical and optical properties were well maintained. Barreau et al*.* realized MoS_2_ film with excellent electrical properties on NaCl substrate [130]. The MoS_2_ obtained by this group is also textured and thus has a photoconductive effect. Unlike Li et al*.*, Barreau et al*.* pre-deposited metal Mo film as a precursor, so the thickness of MoS_2_ film can be controlled by modifying the thickness of the Mo film. It is worth noting that the NaCl substrate can reduce the purity and uniformity of the MoS_2_ film under certain conditions, which is manifested in the following two points: i) The melting temperature of NaCl is about 1123 K. When the actual growth temperature is close to or exceeds this temperature, the substrate softening or even boiling can cause film quality degradation. ii) Na^+^ may pass through MoS_2_ to reach the film surface and react with S atoms to form NaS_x_ (x ≥ 3). However, Na^+^ has been found to have an affinity for H_2_O and O_2_, thus promoting the oxidation of MoS_2_.

Some studies have shown that annealing MoS_2_ film in the S atmosphere can etch residual Na + , and the etching effect increases with time. Therefore, strictly controlling the annealing time to balance the influence of Na^+^ is the key to obtaining high-quality MoS_2_ films. Besides the growth of the MoS_2_, recently, Huan et al*.* used NaCl substrate as a growth template and managed to obtain high-yield preparation of TaS_2_ and NbS_2_. After that, they realized the non-destructive and green transfer of TaS_2_ by taking advantage of the water-soluble characteristics of NaCl. They applied the transferred film to the hydrogen evolution reaction and tested its Tafel slopes, which fluctuate between 61–80 mV dec^−1^ and are equal to the value of Co-doped MoS_2_ foam.

**Molten metal**

TMDCs film grown on rigid and inert substrates often has problems such as an uneven number of layers, small domain size, and high concentration of defect (S vacancies, anti-site defects, impurity atoms, and dislocations), which in turn induces a localized state in band gap and arise hopping transport behavior to decrease carrier mobility. Rigid and inert substrates are not compatible with the manufacturing process of flexible devices, which cannot further expand the application of the film. Taking sapphire and mica, for example, their six-fold symmetry does not match the three-fold symmetry of TMDCs materials, which easily leads to the generation of antiparallel domains (0° and 60° orientation) and twin grain boundaries. Crystal domains of different orientations merge to form a polycrystalline film. In this mirror, a double crystal boundary exhibits metallic characteristics and acts as a conductive channel, which can reduce the photoelectric properties of the film.

Since the surface symmetry of the substrate is determined by the periodicity of its crystal lattice, an amorphous substrate (liquid metal) can be used to break the limitation of substrate symmetry on film growth [84, 106, 131]. There have been experiments to grow self-aligned graphene with liquid Cu and h-BN on liquid Au [132]. In the latter, the solubility of B atoms and N atoms in liquid Au is low. They can diffuse fully and form tightly packed circular crystal domains, which are then rotated to the same orientation by electrostatic interaction and merged into centimeter-level (3 cm) single-crystal h-BN. Due to the low evaporation pressure and the low melting point of Ga, Cao et al*.* reported the growth of layered MoP single crystal on the liquid Ga surface [133]. The surface of liquid Ga has an atomic plane surface, facilitating it's nuclear and growth. By bubbling the gas in liquid Ga, Zavabeti et al*.* reported the growth of layered metal oxides, such as HfO_2_, Al_2_O_3_, and Gd_2_O_3_, which enlarge the two-dimensional family [134].

#### Substrate Pre-treatment

As the growth of the TMDCs would be realized by adsorption and self-assembly of the molecular or cluster, thus the growth substrate surface plays a vital role during the nuclear and growth. During the investigation, it was found that nuclear is still a challenge, and the different groups reported different growth mechanisms. For example, it was found that the pure cleaning of the growth substrate would facilitate its growth. However, the pre-treated growth substrate, such as etching pit on the surface or spin-coating with seed, would benefit its growth.

##### Patterning and Step Creating

**Fig. 9:**
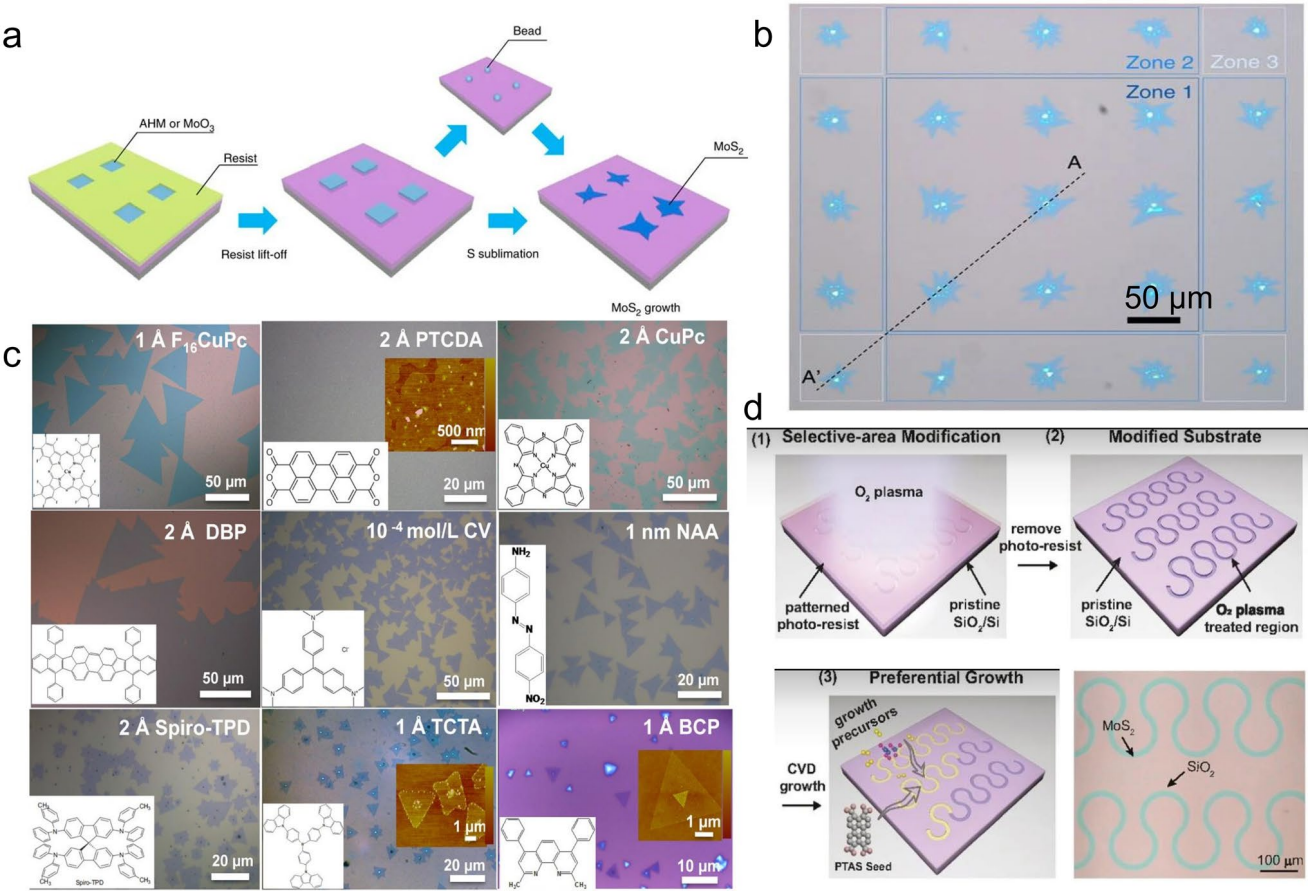
Growing MoS_2_ by using the treated substrate. **a**, **b** Precursor has been pre-patterned on the growth substrate [125], Copyright 2015, Springer Nature. **c** the influence of organic seeding promoter on the MoS_2_ growth [27], Copyright 2014, American Chemical Society. **d** the organic molecular is pre-patterned on the growth substrate [83], Copyright 2019, National Academy of Sciences

It has been reported that the nucleation energy barrier of graphene at the edge of the step is significantly reduced. Therefore, Najmaei et al*.* proposed that a similar edge catalysis process may also exist in the initial stage of MoS_2_ growth [14]. Through deliberate manufacturing defects, it was found that the triangular MoS_2_ film aggregated and grew near the edge of the substrate, causing scratches and dust particles. Thus, a step edge can be created on the substrate surface through a photolithography process to increase the formation of MoS_2_ seeds. The surface of the Si/SiO_2_ substrate would be treated with a uniformly distributed rectangular SiO_2_ columnar area, which increased the nucleation density and allowed isolated crystal domains to merge into a continuous film. By a similar method, Zheng et al*.* reported the growth of the HfS_2_ by using the pre-pattered growth substrate [135]. Through pre-pattern and forming the island of MoO_3_ or ammonium heptamolybdate (AHM) precursor on the growth substrate, Han et al*.* reported the growth of a high-quality single-layer crystal MoS_2_ with carrier mobility of 10 cm^2^/Vs through photolithography as shown in Fig. 9a, b [125]. Using a pre-patterned substrate as a growth template, Guo et al. realized the growth of a single-layer MoS_2_ with a spatial resolution of 2 μm and with carrier mobility of 30 cm^2^ V^−1^ s^−1^ and an on–off ratio of 10^7^, as shown in Fig. 9d [83]. Under the condition that the growth template is unchanged, using a negative photoresist can obtain a MoS_2_ pattern opposite to the patterned area. In addition to photolithography, O_2_ plasma treatment can also create areas with high surface energy so that MoS_2_ film preferentially grows in the treated areas. In practical applications, surface treatment often works with hydrophilic salts such as PTAS to promote the size growth of MoS_2_ films, which can be attributed to the fact that PTAS drives more precursors to attach to selective regions. Guo et al*.* demonstrated a similar contact angle between the treated area and the original area, eliminating the interference of physical roughness by Ar plasma treatment [83]. They found that O_2_ plasma treatment increased the surface energy of the treated area, which showed that the O bond at unsaturated sites in the treated region formed an O-rich surface.

The patterning process and step creating are based on simple photolithography technology [14, 125], which not only guarantees the integrity of the film structure but also simplifies the subsequent device manufacturing process and can be applied to various film and substrate combinations.

##### Seed promoter

Seed promoters have advantages in promoting nucleation, increasing yield, and good repeatability. Crystal violet (CV) is a kind of staining solution commonly used in tissue or cell staining, which can dye the nucleus dark purple. As one of the seed promoters, CV can catalyze the nucleation and growth process of transition metal chalcogenides. Ko et al*.* took triangular CV with both polar and non-polar ends as seed promoters and studied its mechanism in promoting monolayer MoS_2_ growth [58]. The results show that the geometric configuration of CV plays a significant role in modifying the direction (horizontal/vertical) of the MoS_2_ crystal. And the change in the solution polarity can affect CV's geometric configuration on SiO_2_/Si substrate. The geometric configuration of CV is summarized as follows: (i) Polar end concentrated in the center of CV micelle; (ii) Non-polar end concentrated in the center of CV micelle; (iii) Randomly distributed CV molecules. It was found that monolayer MoS_2_ domains mainly grow randomly distributed lying-down CV configurations, while multi-layer MoS_2_ crystal appears in concentrated polar parts in CV micelle. According to density functional theory, this phenomenon can be attributed to the preferential adsorption of S atoms to the polar part of the CV. The adsorption of CV on the SiO_2_/Si substrate is mediated by its polar region. It is further enhanced by lying-down configuration, which can significantly promote the growth of the monolayer MoS_2_ due to the enhanced thermal stability and the ability to prevent CV from reassembling at high temperatures. In addition, the lying-down configuration can achieve more vital CV-SiO_2_ interaction. Therefore, a randomly-distributed lying-down configuration provides optimal conditions for the monolayer MoS_2_, especially when Cl^−^ is placed between CV and substrate.

It is reported that the mixed homogeneous solution of an organic promoter (metalloporphyrin) and the water-soluble metal precursor supports the growth of large-scale intrinsic and doped MoS_2_ films. Organic accelerators are suitable for liquid-phase precursors and solid-phase precursors, having the advantage of being widely used. In addition, an organic promoter has apparent catalytic effects, which can lower the reaction temperature and reduce the residue of amorphous carbon. Presently, perylene-based compounds, namely per 3,4,9,10-tetracarboxylic dianhydride (PTCDA) and per-3,4,9,10-tetracarboxylic acid tetra potassium salt (PTAS), are considered the best organic accelerator [38, 56]. PTAS can also ensure outstanding optical quality and carrier mobility. In a sulfuration reaction, PTAS can preferentially induce the precursor to adsorb to the pretreatment area. Guo et al*.* treated SiO_2_/Si substrate with O_2_ plasma, and the treated area showed a specific pattern [83]. Due to its hydrophilicity, PTAS prefers the patterned area, which results in higher adsorption energy of Mo-containing precursors in this area, enabling selective growth of MoS_2_ film. It is worth noting that O_2_ plasma and PTAS have a synergistic effect on the growth of MoS_2_ film. Regarding the mechanism of alkali metal halides on film growth, apart from the above explanation about the Na_2_S_x_ chain, Kim et al*.* demonstrated that it is similar to planar ring seed promoters, such as PTAS, PTCDA, F_16_CuPc, etc.[49]. The mechanism may involve the energy of the surface/interface/edge of the nucleus, surface diffusion rate, rate of active substance attachment, and density of preferential nucleation sites.

Based on previous reports [125], coating materials containing carbon ring structure and stable at MoS_2_ growth temperature (perylene-3,4,9,10-tetracarboxylic dianhydride, perylene-3,4,9,10-tetracarboxylic acid tetra potassium salt or reduced graphene oxide) on the surface of SiO_2_/Si substrate can promote the expansion of film's lateral size. Lee et al*.* found that rGO can assist in merging adjacent films and excluded GO interference [136]. Li et al*.* analyzed and compared the promotion effects of several seed molecules [83]. The results showed that the side length of triangular MoS_2_ with PTAS-assisted growth could reach 70 μm. However, CuPc and CV were also used as seed promoters, and the film size was only 20 μm, so PTAS has the best auxiliary effect. The insignificant promoting effect of the other two promoters can be attributed to their lower sublimation or decomposition temperature, thus weakening the catalytic effect. Regarding the perspective of electrical performance, the auxiliary product of PTAS is still better than the others. The carrier mobility of PTAS-assisted monolayer MoS_2_ film is 23.2 cm^2^ V^−1^ s^−1^, and the on–off ratio is 10^6^–10^7^, much higher than the corresponding value of a mechanically exfoliated sample. The carrier mobility of CuPc-assisted and CV-assisted single-layer MoS_2_ is 4.2–7.6 and 1 cm^2^ V^−1^ s^−1^, respectively. The threshold voltage of the latter shows a significant negative shift, indicating that there are a lot of structural defects inside. In addition to the above three seed promoters, some aromatic molecules have a similar promoting effect, which can assist the direct growth of MoS_2_ film on various hydrophobic substrates through thermal evaporation deposition. Since PTAS is challenging to adhere to the surface of hydrophobic substrates uniformly, Ling et al*.* found that F_16_CuPc can replace it and promote the growth of large-scale, high-quality, and uniform monolayer MoS_2_ on hydrophobic substrates, as shown in Fig. 9c [27]. Furthermore, the researchers demonstrated that the promotion effects of various seed molecules on the growth of MoS_2_ is in order of (CuPc, PTCDA, DBP, CV) > (NAA, spiro-TDP, TCTA) > (BCP, TPBi, spiro-2-NPB, Ir(ppy)_3_). Chen et al*.* changed the dispersion behavior of precursor on SiO_2_/Si substrate through salinization of the substrate and synthesized a wafer-scale single-layer WS_2_ film [137]. The photodetector based on it showed excellent photoelectric performance (high responsiveness of 3.07 A W^−1^ and quantum efficiency of 763%). Some studies on salinization treatment pointed out that its mechanism may be similar to seed promoters.

##### Cleaning

Compared with the post-annealing process, which can reduce defect concentration and improve the quality of film crystallization, the pre-annealing treatment could tune the structure of the growth substrate, including the roughness, contamination, and recrystallization et al*.* Recently, Andrzejewsk et al*.* showed that pre-annealing substrate surface could also effectively improve domain size of MoS_2_ film [45]. They used an H_2_ atmosphere to heat the growth substrate in advance and got a MoS_2_ film with a grain size twice the previous results. Besides, wet chemical cleaning has also been widely used. Conventional cleaning methods, including HF treatment, piranha solution treatment, precursor dip-coating, silanization treatment, etc., have been carried out. The purpose is to obtain a hydrophilic surface, thereby improving the uniformity of nucleation or the dispersion of precursors. Li et al*.* used a standard piranha solution to treat SiO_2_/Si substrate. They successfully obtained a few layers of MoS_2_ film with high crystallinity and uniformity, whose carrier mobility and on–off ratio are about 6 cm^2^ V^−1^ s^−1^ and 10^5^, respectively.

#### Other Factors

Besides the above factors, there are some factors whose effects are less than those mentioned, and the general influence can vary from case to case. The first one is precursor concentration. According to classical crystal growth theory, film morphology is controlled by the precursor concentration on a substrate surface. Generally speaking, the larger the precursor concentration, the larger the film size. The smaller the precursor concentration fluctuation, the more uniform the film thickness. Yu et al*.* used an improved CVD device to increase the local concentration of precursor and obtained a single-layer triangular WS_2_ film with a side length of 178 μm [18]. By exploring the reaction mechanism, they found that the reactant atoms first form triangular WS_2+x_ flakes, which can be used as nucleation sites to adsorb more WS_2+x_, so that a series of overlapping triangular flakes continue to adsorb at this site, then being reduced in S atmosphere and finally transformed into a regular triangular film.

The second one is growth temperature. It has been reported that growth temperature can control the orientation and size of MoS_2_ film [18, 20]. Under the condition that the interface between substrate and film has rotational anisotropy, a lower growth temperature is more conducive to the alignment of seed crystals, which can be attributed to the fact that high temperature can provide higher energy. The seeds are more inclined to random orientation. Based on Sial et al*.*'s results, the film's size increases with the growth temperature (from 4 to 50 μm) [56]. When the growth temperature is lower than about 725 °C, the film mainly exhibits a few layers. When the temperature is increased to about 800 °C, a large number of MoO_3_ molecular clusters on the substrate surface are selenized, forming island-shaped MoO_3-x_Se_y_ nanoparticles. Once the temperature was raised to 850 °C, the previous nanoparticles evolved into triangular MoSe_2_ film with multiple layers in the center. It is worth noting that the morphology of the film does not change with the growth temperature and is primarily triangular. However, the change in the precursor's flux may cause the terminal edge's chemical composition to fluctuate. According to classical crystal growth theory, an edge with a slower growth rate determines the film's shape and the end edge's chemical composition.

In addition, a few studies have proved that prolonging sulfuration time can affect the size of MoS_2_ film. This can be attributed to the fact that solid precursor is easy to evaporate in a low-pressure environment, thereby increasing the precursor flux in reacting chamber and promoting a large-scale monolayer film growth. However, this conclusion can be controversial, as Xu et al. [138] and Chang et al. [74] found that the growth rate in vertical is sensitive to reaction time, and this regulation is liable to obtain MoS_2_ film with few-layer (1–3 L). This can be attributed to the fact that introducing S vapor later is conducive to forming large droplets, encapsulating small MoS_2_ seeds in a supersaturated solution during the subsequent sulfuration process. In this case, when single-layer MoS_2_ film is grown, another layer continues to grow on the edge of the original seed. Interestingly, when the film thickness is close to 3 layers, the process can hardly be continued only by delaying time.

#### Brief Sum-Up

**Fig. 10:**
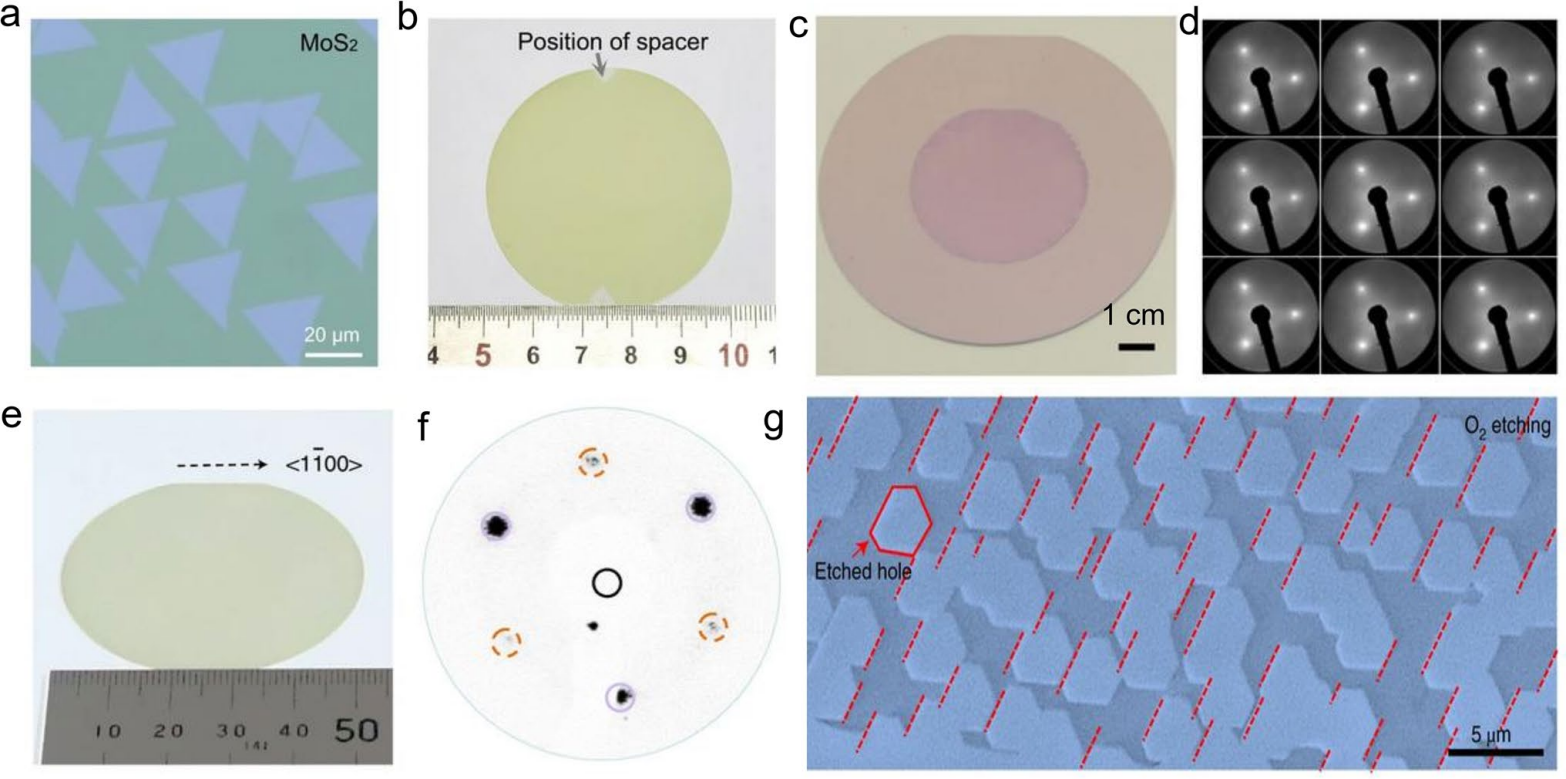
The wafer-scale growth of MoS_2_ and WS_2_ by CVD method. **a**, **b** 2-inch polycrystalline MoS_2_ film [139], Copyright 2022, Springer Nature. **c**, **d** 2-inch single-crystal monolayer MoS_2_ film and its LEED patterns over a 1 cm^2^ area [108], Copyright 2021, Springer Nature. **e**–**g** 2-inch single-crystal monolayer WS_2_ film and the representative LEED pattern [140], Copyright 2022, Springer Nature

Among all the synthesis methods, CVD is the most compatible with existing semiconductor technology and can precisely control the parameters required for the growth of atomic-level film in theory. Typically, it directly forms 2D materials on the substrate, and transfer can be omitted in some cases. As shown in Fig. 10, using active chalcogen monomer from ZnS crystal, Liu et al*.* reported the 2-inch polycrystalline monolayer MoS_2_ film, where its electronic mobility is ~ 42 cm^2^ V^−1^ s^−1^ [139]. Wang et al. optimized the growth substrate to have the miscut orientation toward the A axis (C/A) of sapphire. This substrate's epitaxial growth of a 2-inch single-crystal monolayer MoS_2_ film is realized. Its electron carrier mobility can rise to 102.6 cm^2^ V^−1^ s^−1^, and the saturation current reaches 450 μA μm^−1^ [108]. At the same time, Liu et al*.* also reported the growth of a 2-inch single-crystal monolayer WS_2_ film by epitaxially growth on a sapphire substrate [140]. As shown in Table 1, it can be seen that the size of the TMDCs film is strongly dependent on the growth parameter. However, it is susceptible to growth conditions, thus resulting in the domain size fluctuating in a wide range, from a few to hundreds of microns. It is necessary further to reduce the influence of the environment on resulting materials. In addition to direct control methods discussed in this article, such as raw material replacement and substrate treatment, growth parameters can also be indirectly affected by the improvement of the traditional CVD device. Unquestionably, large-area growth of highly uniform MoS_2_ will enable the future batch fabrication of the new generation of devices and circuitry.

**Table 1:** Summarized the growth parameter and its property of MoS_2_ et al. typical 2D semiconductor

Materials	Growth method	Pressure (Torr)	Mo source	S source	Gas	Catalysis	Promoter	Growth substrate	Substrate pre-treatment	Layer	Structure	Size	Carrier mobility (cm^2^ V^−1^ s^−1^)	Current ON/OFF ratio	References
MoS_2_	Thermal conversation	5–10	MoO_2_	S	Ar	–	–	Al_2_O_3_	–	1	–	2 inch	10 ~ 30	10^3–7^	[37]
MoS_2_	Thermal conversation	1	(NH_4_)_2_MoS_4_	S	Ar/H_2_	DMF	–	SiO_2_/Si	Strong oxidizing solution	3	PC	1 cm	6	1.6 × 10^5^	[31]
MoS_2_	Thermal conversation	75–300	MoO_3_	S	N_2_	–	Pattered SiO_2_	SiO_2_/Si	–	ML	PC	100 μm	4.3 ± 0.8	6 × 10^6^	[14]
MoS_2_	Thermal conversation	NA	Mo(CO)_6_	S	Ar	–	–	Al_2_O_3_	–	1	SC	2 inch	0.76	1 × 10^4^	[28]
MoS_2_	Thermal conversation	1 × 10^–7^	Mo rod	CS_2_	–	–	–	SiO_2_/Si	–	1	PC	1 cm	–	–	[78]
MoS_2_	Thermal conversation	NA	MoO_3_	S	Ar	–	–	SiO_2_/Si	–	1	SC	20 μm	0.1–0.7	10^4^–10^6^	[30]
MoS_2_	Thermal conversion	NA	MoO_3_	S	N_2_	–	–	SiO_2_/Si	Organic solution	1	PC	1300 μm^2^	10	10^6^	[125]
MoS_2_	Thermal conversion	30	Mo(CO)_6_	S	Ar/H_2_	NaF	–	Al_2_O_3_	Organic solution	1	SC	450 μm	49	5 × 10^8^	[74]
MoS_2_	Thermal conversion	NA	Mo film	S	N_2_	–	–	SiO_2_/Si	–	1	PC	1 cm	0.04	–	[33]
WS_2_	Thermal conversation	NA	WH_2_(iPrCp)_2_	H_2_S	Ar	–	–	SiO_2_/Si	–	1	–	1 cm	3.9	–	[141]
MoS_2_	CVD	NA	(NH_4_)_2_MoO_4_	S	Ar	NaOH	–	Al_2_O_3_	Piranha solution	1	SC	200 μm	~ 30	–	[107]
MoS_2_	CVD	760	MoO_3_	S	Ar	–	–	molten glass	Organic solution	1	SC	–	12	10^8^	[112]
MoS_2_	CVD	760	MoO_3_	S	Ar	–	PTAS	SiO_2_/Si	Strong oxidizing solution	1	–	60 μm	–	–	[27]
MoS_2_	CVD	760	MoO_3_	S	Ar	–	–	Al_2_O_3_	Strong oxidizing solution	1	PC	1 cm	70	10^8^	[138]
MoS_2_	CVD	760	MoO_3_	S	N_2_	–	–	SiO_2_/Si	Strong oxidizing solution	1	SC	120 μm	8	10^7^	[89]
MoS_2_	CVD	760	MoO_3_	S	N_2_/H_2_	–	–	SiO_2_/Si	–	1	SC	500 μm	–	–	[88]
MoS_2_	CVD	40	MoO_3_	S	Ar	–	–	Al_2_O_3_	–	1	SC	30 μm	–	–	[87]
MoS_2_	CVD	NA	MoO_3_	S	N_2_	–	rGO-N_2_H_4_	SiO_2_/Si	–	1	PC	2 mm	0.02	10^4^	[136]
MoS_2_	CVD	760	MoO_2_	S	Ar	NaBr	–	SiO_2_/Si	Piranha Solution	1	PC	500 μm	48.7	10^7^	[95]
MoS_2_	CVD	760	MoO_3_	S	Ar/H_2_	–	–	Al_2_O_3_	–	1	PC	4.7 cm	0.033	5.8 × 10^5^	[94]
MoS_2_	CVD	760	MoO_3_	S	Ar	–	–	SiO_2_/Si	–	1	SC	64 μm	–	–	[114]
MoS_2_	CVD	0.25	MoO_3_	S	Ar	–	–	Al_2_O_3_	–	1	PC	100% cover	1	10^6^	[111]
MoS_2_	CVD	NA	Mo foil	S	Ar/O_2_	–	–	Molten glass	DI water	1	SC	400 μm	11.4	10^6^	[66]
MoS_2_	CVD	0.570	Mo film	MoS_2_	Ar	–	–	SiO_2_/Si	Plasma	1	PC	1 mm	0.05	10^4^	[29]
MoS_2_	CVD	NA	MoCl_5_	DMS	Ar/H_2_	NaCl	–	SiO_2_/Si	–	1	SC	50 μm	10.4	3 × 10^7^	[103]
MoS_2_	CVD	0.25	MoO_3_	S	Ar	–	–	Au foil	–	1	SC	50 μm	–	–	[123]
MoS_2_	CVD	760	MoO_3_	S	Ar	–	PTAS	SiO_2_/Si	Plasma	1	PC	Pattered	29.3	10^7^	[83]
MoS_2_	CVD	760	MoO_3_	S	Ar	–	–	SiO_2_/Si	–	1	SC	305 μm	30	10^6^	[15]
MoS_2_	CVD	NA	Mo foil	S	Ar	–	CV	SiO_2_/Si	UV ramp	1	SC	20 μm	–	–	[58]
WS_2_	CVD	10	WO_3_	S	Ar	NaCl	–	Al_2_O_3_	Piranha solution	1	SC	1.7 mm	11	10^3^	[142]
WS_2_	CVD	0.35	W foil	S	Ar/O_2_	NaOH	–	Al_2_O_3_	–	1	SC	550 μm	1.21	1 × 10^6^	[93]
WS_2_	CVD	NA	(NH_4_)_6_H_2_W_12_O_40_	H_2_S	N_2_	–	–	Au foil	Annealing	1	SC	420 μm	20	10^8^	[72]
WS_2_	CVD	760	WO_3_	S	Ar	–	–	Au foil	Annealing	1	SC	1 mm	1.7	5 × 10^7^	[96]
WS_2_	CVD	NA	WO_3_	S	Ar	–	–	SiO_2_/Si	–	1	SC	50 μm	–	–	[55]
MoSe_2_	CVD	30	MoO_3_	Se	H_2_/N_2_	NaCl	–	SiO_2_/Si	–	1	SC	250 μm	38	10^5^	[46]
MoSe_2_	CVD	NA	MoO_3_	Se	Ar/H_2_	–	–	SiO_2_/Si	–	1	SC	700 μm	42	10^6^	[86]
MoSe_2_	CVD	20	MoO_3_	Se	H_2_	–	–	SiO_2_/Si	–	1–2	PC	1 cm	0.02	~ 10^2^	[113]
MoSe_2_	CVD	760	MoO_3_	Se	Ar/H_2_	–	PTAS	SiO_2_/Si	–	1	SC	50 μm	–	–	[56]
MoSe_2_	CVD	760	Mo foil MoO_3_	Se	Ar/H_2_	–	–	molten glass	Organic solution	1	SC	2.5 mm	95	10^7^	[92]
WSe_2_	CVD	1	WO_3_	Se	Ar/H_2_	–	–	Al_2_O_3_	–	1	SC	50 μm	90	10^6^	[59]
NbSe_2_	CVD	760	NbOx(x ≤ 2.5)	Se	Ar/H_2_	NaCl	–	SiO_2_/Si	–	1	SC	200 μm	Superconductor		[97]
MoS_2_	MBE	4.0 × 10^–5^	MoO_3_	S	–	–	–	Graphene	–		PC	–	–	–	[85]
MoS_2_	MOCVD	7.5	Mo(CO)_6_	(C_2_H_5_)_2_S	Ar/H_2_	–	–	SiO_2_/Si	–	1	PC	4 inch	29	10^6^	[47]
MoS_2_	MOCVD	0.700	Na_2_MoO_4_	(C_2_H_5_)_2_S	Ar/H_2_	–	–	SiO_2_/Si	KOH	1	PC	2 inch	21.6	–	[48]
MoS_2_	MOCVD	NA	Mo(CO)_6_	H_2_S	Ar	KI、NaCl	–	Al_2_O_3_	Pre-annealing	1	SC	10 μm	100	–	[49]
WSe_2_	PVD	NA	WSe_2_	WSe_2_	Ar	–	–	SiO_2_/Si	–	1	SC	800 μm	90	2 × 10^6^	[98]
MoS_2_	PVD	0.015	MoS_2_		Ar	–	–	SiO_2_/Si	Organic solution	3–6	PC	1 m^2^	–	–	[143]

*Al*_*2*_*O*_*3*_ means the sapphire substrate, *SC* means the single-crystalline, *PC* means the polycrystalline

## Applications of MoS_2_ in Integration Circuits

**Fig. 11:**
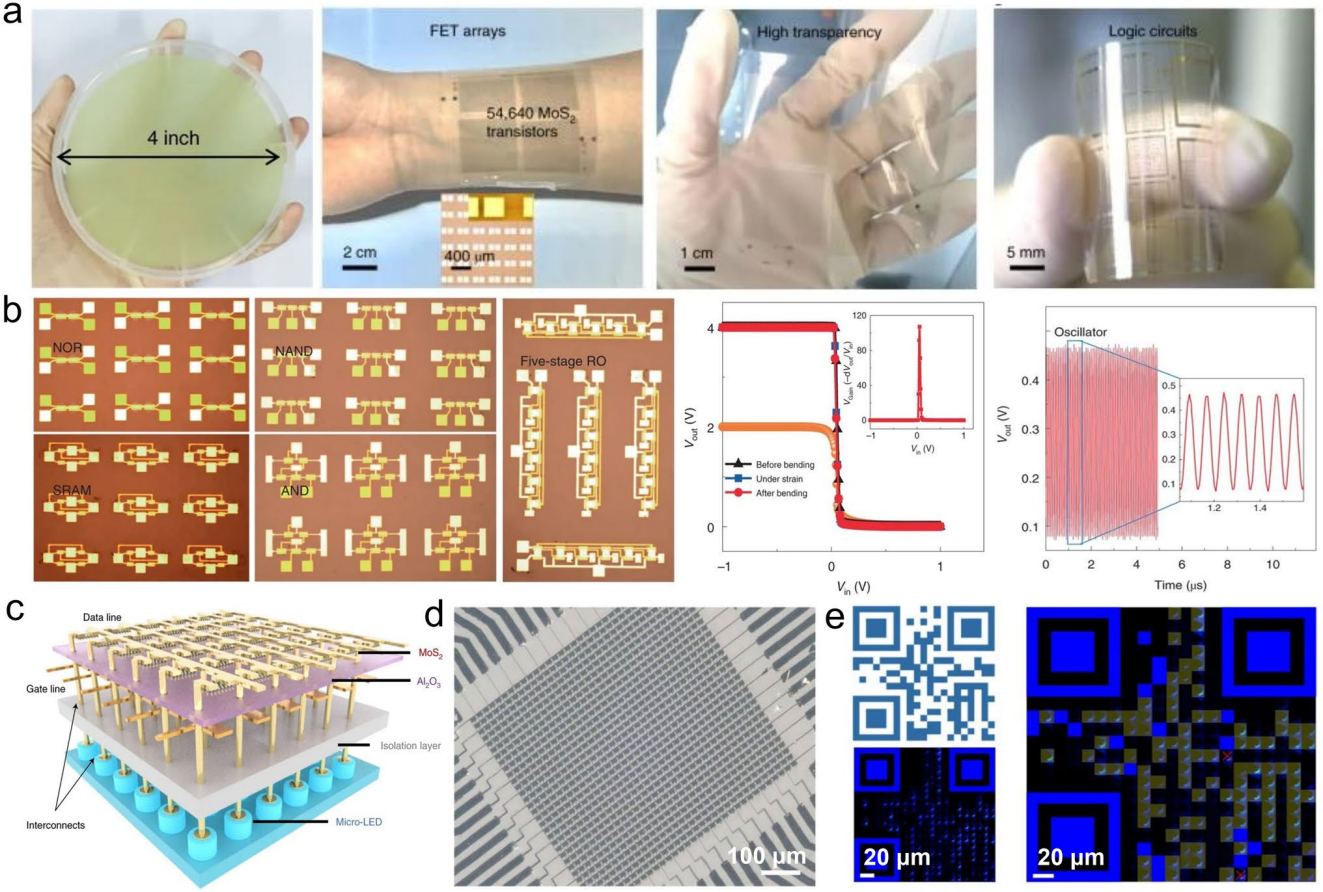
Application of MoS_2_ film in integrated circuits. **a** The wafer-scale growth of MoS_2_ and its optical image of flexible devices, **b** The circuits of MoS_2_-based NOR, NAND, SRAM, AND and Five-stage RO circuits and the performance of inverters and oscillators [3], Copyright 2020, Springer Nature. **c**, **d** The diagram and optical image of the MoS_2_ transistor derived micro-LED display, **e** The images of the QR code displayed on a 1270-PPI blue micro-LED display [154], Copyright 2021, Springer Nature

As discussed above, it can be seen that the wafer-scale growth of MoS_2_ already gets much investigation. The MoS_2_ film grows from nanometer to 2-inch in single-crystal structure and 4-inch in polycrystalline structure, as shown in Fig. 11a, b [3, 108, 140]. Due to its well physical and chemical properties, it has been proofed showing excellent potential applications in such as a transistor, logical device, photodetector, gas sensor, catalysis et al. [144] By optimizing the metal contact electrode, the carrier mobility of monolayer MoS_2_ can rise to 167 cm^2^ V^−1^ s^−1^ [2, 23]. The MoS_2_ transistor with ultra-short length also shows typical electric field modulation [4, 145]. Its photoresponsivity can be significantly enhanced by integrated ferroelectric materials or by forming an AA-stacking configuration [101, 146]. When employing MoS_2_ as the sensitive material, it was found that the detection limits can be improved in CO, CO_2_, and NO_x_, etc. gases [147–150]. Therefore, it is likely that the MoS_2_ will be one of the most potential applications in real life. However, up to now, these potential applications have been performed on the mechanically exfoliated MoS_2_ flake or micrometer-size grown MoS_2_. Thus, the real application of wafer-scale MoS_2_ is still in its early stage. In the following sections, we will summarize the wafer-scale application of MoS_2_ in a transistor, inverter, electronics, and photodetectors.

### Transistors, Inverter Electronics

Transition metal chalcogenides represented by molybdenum disulfide can be used to manufacture a wide range of emerging electronic devices. Among them, suspended 2D nanostructures can improve the performance of nanoelectronic devices through the electronic coupling of advanced substrate effects to be applied to practical applications such as sensing [151, 152]. Wang et al*.* constructed logic circuits such as logic inverters, NAND gates, and static random-access memory (SRAM) based on double-layer MoS_2_ film and demonstrated the great potential of 2D materials in improving device performance and giving new functions to electronics and display technology [153]. From the voltage transmission curve, when the input voltage *V*in = 2 V, the conductivity of E-type MoS_2_-FET is significantly higher than that of D-type MoS_2_-FET. However, when the input voltage *V*in = 0 V, the transistor does not turn on. At the same time, information about the voltage gain of the inverter circuit can also be obtained. A voltage gain of nearly 5 can be achieved in this experiment. Besides, the authors also demonstrated its potential application in the NAND gate circuit. Taking the NAND gate circuit as a function of time to record the output voltage, it is not difficult to find that the double-layer MoS_2_ field-effect transistor has great application potential in the field of NAND gate functional circuits. Furthermore, as one of the most basic logic gates, the NAND gate can be combined with the right and wrong gates to build more complex logic gates, highlighting the possibility of MoS_2_'s application in digital integrated circuits.

By the CVD method, Liu et al. recently reported the 2-inch bilayer MoS_2_ and found that the short-channel FETs exhibit an on-state current of 1.27 mA μm^−1^, which exceeds the 2028 roadmap target for high-performance FETs [57]. Zhang et al*.* reported the 4-inch growth of MoS_2_ film and fabricated its flexible electronics, as shown in Fig. 11. Combining the back-gated transistor using Al_2_O_3_ as the insulator, the device array has been realized, including inverters, NOR gates, NAND gates, AND gates, static random-access memories, and five-stage ring oscillators. The device density can reach 1518 transistors cm^−2^ and yield 97%. Regarding the five-stage ring oscillators, a stable oscillation frequency of 13.12 MHz corresponding to a stage delay of 7.6 ns is achieved. The performance is comparable to state-of-the-art flexible ring oscillators made of various semiconductor materials. By MOCVD, Wang et al. demonstrated the growth of the 2-inch single crystalline MoS_2_ film. The median mobility of 54 cm^2^ V^−1^ s^−1^ and drive current of 210 μA μm^−1^ is achieved [154]. By using the MoS_2_ transistors to drive the micrometer-sized LEDs, its luminance can reach as high as 7.1 × 107 cd m^−2^ at the voltage of 8 V, and the resolution is about 1270 pixels-per-inch, as shown in Fig. 11c–e.

### Photodetectors

**Fig. 12:**
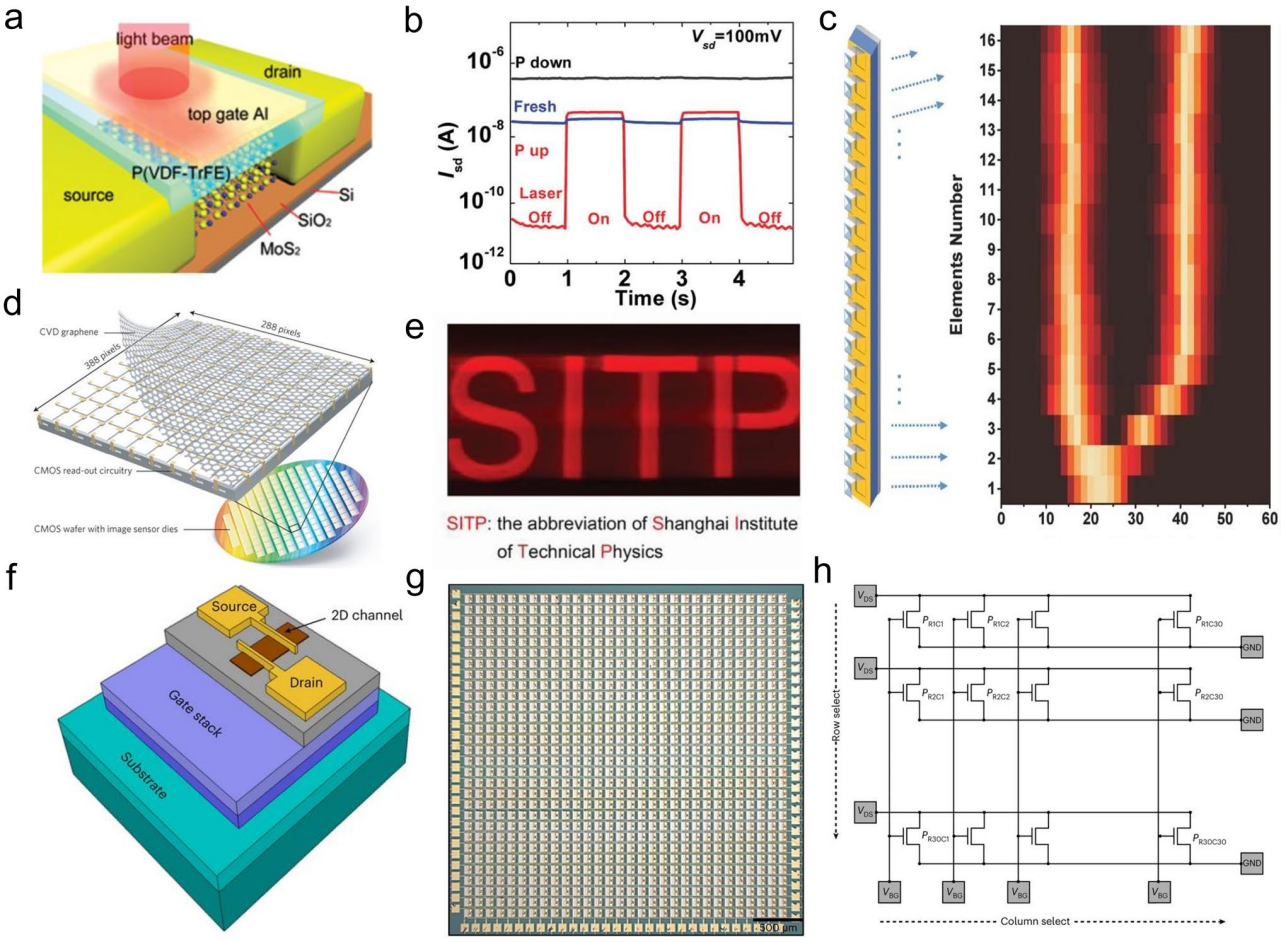
2D materials-based photodetector. **a** Schematic diagram of the ferroelectric enhanced MoS_2_-based photodetector, **b** MoS_2_ current as the function of the ferroelectric polarization [146], Copyright 2015, WILEY–VCH. **c** Graphene-based photodetector array [169], Copyright 2017, Springer Nature. **d** Infrared image obtained from single-pixel imaging setup prepared by the MoS_2_ photodetector [155], Copyright 2019, WILEY–VCH. **e** The infrared image obtained from linear array imaging (16 × 60 pixels) by using GaSe/GaSb heterostructures [170], Copyright 2017, WILEY–VCH. **f–h**) The monolayer MoS_2_ phototransistor-based active pixel sensor array [168], Copyright 2022, Springer Nature

In recent years, two-dimensional atomic crystals have shown rich physical properties due to their atomic thickness, high carrier mobility, and easily adjustable electronic structure, which makes them essential research value not only in the discussion of physical mechanisms but also in the design and potential application of devices. In the field of photoelectric detection, on the one hand, the channel volume of optoelectronic devices based on two-dimensional atomic crystals will be significantly reduced (the thickness of the absorption layer is about 1 ~ 5 nm), and the intrinsic dark current proportional to the device volume will be significantly reduced [155–157]. At the same time, after the longitudinal transport is limited, the carrier is easily regulated by the local field in the transverse transport, and photoelectric detection with a high signal-to-noise ratio is expected to be realized at room temperature. On the other hand, two-dimensional atomic crystals have very high intrinsic photoelectric gain (10^3^ ~ 10^8^), which breaks through the theoretical limit of the photoelectric response of traditional thin film materials and is expected to obtain a high photoelectric response [158]. Therefore, infrared detection based on two-dimensional atomic crystals is expected to achieve high sensitivity at room temperature, attracting extensive attention from researchers worldwide in recent years. MoS_2_ et al*.* 2D materials have been widely used as photoresponse materials due to their high light-matter interaction, high carrier mobility, and extensive range of sensing wavelengths. The direct energy configuration of monolayer MoS_2_ would facilitate its photoexcitation resulting in photodetection. However, the high transmittance of the monolayer MoS_2_ is about 92%, which indicates that most phones would directly go through the materials without efficient conversion. Therefore, one strategy is proposed that suppressing the dark current would help to improve the photocurrent resulting in enhanced photoresponsivity. In 2013, Lopez-Scanchez et al. demonstrated the potential application of MoS_2_ in photodetection [159]. It was found that the gate voltage can tune the photoresponse, and a maximum photoresponsivity of 880 A W^−1^ was achieved when the gate voltage was set near the threshold voltage where the dark current was the lowest. Recently, the interface polarization provided by the ferroelectric layer, such as P(VDF-TrFE) and HfZrO, has been employed to construct the 2D materials-based photodetector [146, 160–162]. For example, Wang et al*.* reported that the photoresponsivity of MoS_2_ could be increased to 2570 A W^−1^ when the ferroelectric polarization is turned down. Furthermore, the tuned electronic structure could extend the response range into near-infrared. Besides the pure 2D materials, their van der Waals heterojunctions have also been demonstrated, showing well-tunable photoresponse [16, 163]. Although photoresponsivity is already satisfied the industry requirement, the response time, including the rise time and the fall time, is still challenging. Introducing interfacial molecular such as ZnPc, CuPc, CuInSe_2_, could passivate the MoS_2_, leading the photogenerated hole could effectively separate from the intrinsic trap in MoS_2_ or interfacial trap along the insulator surface, resulting in suppressed persistent photoconductivity effect [164–167]. The life of the photogenerated hole could be effectively shortened, and the response time could improve from seconds to milliseconds or microseconds. However, photoresponsivity has some sacrifices. Even though the 2D materials-based photodetectors have already been proven potential applications in the photodetector, especially infrared imaging, as shown in Fig. 12. As shown in Fig. 12f, a 2D active pixel sensor was recently demonstrated by using a monolayer MoS_2_ phototransistor array. In this sensor, each pixel uses a single programmable MoS_2_ phototransistor. It shows a substantial reduction in footprint of 900 pixels in ∼0.09 cm^2^ and energy consumption of fJ per pixel in 100 s. Furthermore, it exhibits a high dynamic range of ∼80 dB and in-sensor de-noising capabilities, showing the potential application in future high-performance active pixel sensors [168].

## Conclusion and Prospect

Two-dimensional (2D) nanostructured materials represented by 2D-TMDCs are one of the most promising candidates for next-generation electronic and optoelectronic devices in the "post-Moore" era. However, the fabrication technology of large-scale, high-quality 2D-TMDCs materials is still one of the critical bottlenecks to be broken through for final industrial application. This article takes MoS_2_, for example, to provide a comprehensive summary and appreciation of the recent progress in the fabrication of 2D-TMDCs materials, including the synthesis methods, properties, and applications. Specific experimental techniques, such as physical vapor deposition, chemical vapor deposition, are introduced, among which the CVD method is the most compatible with existing semiconductor technology. Thus, the emphasis is on discussing the growth factors such as precursor type, growth pressure, carrier gas, and catalyst effect to summarize the structure and properties of MoS_2_ film prepared based on CVD systems. However, there are still many challenges in the CVD growth of two-dimensional materials. For example, more than understanding the CVD growth mechanism is needed, and it is still necessary to further study the crystal growth kinetics of different materials. Secondly, CVD technology should be further improved in order to synthesize larger areas of continuous films, especially high-quality single-crystal films. Thirdly, device applications have extensive requirements for two-dimensional heterostructures, but it is still challenging to obtain large-area and high-quality two-dimensional heterostructures based on CVD. Finally, based on the current status of CVD research, this paper puts forward the following prospects:

**Growth mechanism**

According to the crystal growth theory, the nucleation size and nucleation barrier of the material under certain conditions can be obtained by combining the crystal nucleation dynamics with the CVD process. As mentioned in this paper, the seed promoter growth of MoS_2_ can realize the large-area growth of a single-grain film at a low chemical potential according to the critical energy required for the growth. An in-depth understanding of the growth mechanism of more materials is conducive to accurately synthesizing large-area 2D materials based on their growth dynamics. In addition, when MoS_2_ is grown on the sapphire surface, the reconstruction of the boundary depends on the strength of the interaction between MoS_2_ and the substrate. The novel boundary structure will significantly change the equilibrium morphology and growth behavior of MoS_2_ grains

**Growth substrate**

To find and design a suitable substrate, the lattice orientation can be determined by the surface symmetry of the substrate, and the operation on the substrate can reproduce the preferential energy orientation of the atomic nucleus. For TMDCs, the synthesis technology of large-size single crystal materials is still in the early research stage, and there are a lot of unsolved problems: especially for insulating substrates (such as mica and sapphire) that are more suitable for TMDCs growth, the existence of antiparallel domains will lead to the formation of twin grain boundaries. The metal precursor has poor wettability on the insulating substrate of some crystal planes and tends to grow vertically, which will seriously affect the preparation of monolayer films and increase the difficulty of growth. Due to the uneven distribution of insulating substrate steps on some crystal planes, the increase of surface barrier will lead to the difficulty of TMDCs nucleation, which also limits the further growth of single crystal films. Choosing a substrate with the exact surface symmetry as two-dimensional materials makes it easier to achieve the oriented growth of TMDCs domains and merge into an integral single crystal film. For example, the sapphire substrate has the exact lattice symmetry as MoS_2_, so sapphire is a common substrate for preparing MoS_2_ films. Currently, 2-inch MoS_2_ single-crystal thin films have been successfully prepared on this substrate.

**Innovation and improvement of the growth system**

The growth of 2D TMDCs usually involves solid precursors, and its concentration is difficult to accurately control. Therefore, it is necessary to develop an advanced CVD system to make material growth more controllable. MOCVD developed at present can stabilize the precursor concentration in the reaction chamber, and PECVD and ICP-CVD can also significantly improve the stability of the reaction environment. In addition, technological innovation is also needed, such as precise control of reaction conditions through the use of lasers, electric fields, or magnetic fields. First, there is no unified theory on the growth mechanism of 2D materials, and there still needs to be more direct and effective evidence. It may be necessary to develop in-situ methods to comprehensively observe the whole process, especially the in-situ observation of the growth process under high-temperature conditions.

The batch preparation of two-dimensional materials is very complex, especially the extension from the laboratory stage to industrialization, which needs to solve many problems. CVD large-scale preparation is to enlarge further the system size of the laboratory in a similar chemical reaction environment, which will have higher requirements for the uniformity of materials, which means that reaction dynamics and hydrodynamic factors need to be fully considered, such as diffusion, surface adsorption, gas-phase reaction, and other processes. The regulation and control of airflow, reactant vapor concentration, temperature, and substrate within the environmental limit need to be more accurate to ensure the yield of products. In addition, the single crystal thin film grown by the CVD method usually produces cracks and folds due to the mismatch of thermal expansion coefficient between the single crystal thin film and the substrate, which will introduce a large number of defects and affect the service life of the product, which is also a problem that needs to be considered in actual production.

In recent years, the research on 2D semiconductor films is fully emerging. The growth method of 2D semiconductor single crystal films with specific layers and the stacking angle may be the most worthy research direction of 2D semiconductors in the future. Therefore, the precise control of the number of layers of large-area single-crystal 2D semiconductors, the development of new 2D material transfer methods, maintaining the physical integrity in a large area, and avoiding the introduction of any impurities or residues are also the core issues that need to be solved in the future. With the continuous improvement of material synthesis methods, large-scale ultra-clean and non-destructive transfer technology and emerging application research will continue to make breakthroughs. 2D materials will have a better future. At present, silicon-based micro/nanodevices have reached the theoretical limit, and the research of new semiconductor materials is imminent. The natural semiconductor advantages of layered MoS_2_ are expected to meet this demand. CVD is very beneficial to the preparation of large-area high-quality MoS_2_ films. With the continuous development and improvement of preparation technology, the quality of MoS_2_ films will be continuously improved, and their applications will also be continuously expanded.
